# A Survey on Industrial Internet of Things Security: Requirements, Attacks, AI-Based Solutions, and Edge Computing Opportunities

**DOI:** 10.3390/s23177470

**Published:** 2023-08-28

**Authors:** Bandar Alotaibi

**Affiliations:** Department of Information Technology, University of Tabuk, Tabuk 47731, Saudi Arabia; b-alotaibi@ut.edu.sa

**Keywords:** Internet of Things, fog computing, edge computing, Industrial Internet of Things, Industry 4.0, cyber-physical systems, cybersecurity

## Abstract

The Industrial Internet of Things (IIoT) paradigm is a key research area derived from the Internet of Things (IoT). The emergence of IIoT has enabled a revolution in manufacturing and production, through the employment of various embedded sensing devices connected by an IoT network, along with a collection of enabling technologies, such as artificial intelligence (AI) and edge/fog computing. One of the unrivaled characteristics of IIoT is the inter-connectivity provided to industries; however, this characteristic might open the door for cyber-criminals to launch various attacks. In fact, one of the major challenges hindering the prevalent adoption of the IIoT paradigm is IoT security. Inevitably, there has been an inevitable increase in research proposals over the last decade to overcome these security concerns. To obtain an overview of this research area, conducting a literature survey of the published research is necessary, eliciting the various security requirements and their considerations. This paper provides a literature survey of IIoT security, focused on the period from 2017 to 2023. We identify IIoT security threats and classify them into three categories, based on the IIoT layer they exploit to launch these attacks. Additionally, we characterize the security requirements that these attacks violate. Finally, we highlight how emerging technologies, such as AI and edge/fog computing, can be adopted to address security concerns and enhance IIoT security.

## 1. Introduction

The Internet of Things (IoT) can be defined as a paradigm that utilizes intelligent devices that can communicate through the internet [1,2]. IoT environments comprise many intelligent devices capable of collecting, processing, transmitting, and receiving data from each other [3]. These interconnected intelligent devices help us to monitor any environment and precisely control any setting [4]. By 2025, the total economic impact derived from IoT technology annually is predicted to reach USD 11.1 trillion [5]. As most of the IoT systems developed so far are consumer-centric, their nature has enabled the adoption of this technology in many industrial applications, creating the so-called IIoT technology [6]. IIoT, also known as industrial internet, can be defined as a paradigm that utilizes interconnected intelligent devices deployed in an industrial environment, in order to connect industrial components, including actuators, sensors, controllers, and intelligent control systems (i.e., for data analysis and industrial process optimization to enhance the speed of execution, decrease costs, and manage the industrial setting dynamically) [7].

**Figure 1:**
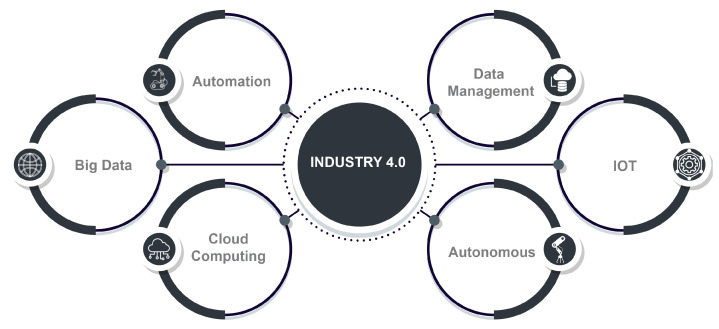
Industry 4.0 utilizes various emerging technologies to improve industrial production.

As shown in Figure 1, Industry 4.0—also known as the fourth Industrial Revolution—exemplifies an unprecedented industrial evolution and complements various emerging technologies and systems, such as CPS, MCC, IoT, AI, CC, big data, and fog computing, in order to improve the adequacy of industries, in terms of heterogeneous data support, automation, high production, and integrating knowledge [8,9]. The number of embedded systems utilized in industrial applications has swiftly grown, due to the mounting availability, capability, and affordability of sensors, communication modules, and processes [10]. This has driven more interest regarding the use of IIoT in industrial domains such as smart cities, transportation, healthcare, microgrids, and smart factories, giving rise to Industry 4.0 based on CPS. By 2030, IIoT has been forecasted to be worth USD 7.1 trillion in the U.S. and to exceed USD 1.2 trillion in European countries [11].

Despite all of the advantages of adopting IIoT, IoT security issues represent one of the biggest challenges hindering its perfect utilization. The poor security associated with IoT devices [12,13] makes them vulnerable to cyber-attacks (e.g., IoT devices could be targeted by adversaries to execute devastating attacks, such as DDoS) [14]. Thus, they may be susceptible to various cybersecurity threats, causing IIoT security to become a hot topic in recent years [15]. IoT also relies heavily on the CC to provide the IoT devices with limited capabilities for the desired services [16]; however, this dependency transports diverse vulnerabilities to IoT environments [17].

In this context, an emerging computing technology, known as fog computing, has attracted the attention of the research community [18]. Fog computing is a new paradigm that bridges the gap between CC and IoT by diffusing services and resources on the path between IoT environments and CC [19]. Fog computing has several advantages, which can facilitate the secure deployment of IIoT devices. However, fog computing may also bring some inherited security challenges to the table [20]. This paper presents a review of the security requirements of IIoT, identifies and classifies cyberattacks that target IIoT environments, surveys AI-based solutions that enhance IIoT security, and highlights edge computing opportunities.

The contributions of this paper can be summarized as follows:

- The security requirements and challenges encountered in IIoT environments are highlighted.
- Solutions based on AI to these security challenges are thoroughly investigated.
- Opportunities and challenges for the secure deployment of IIoT devices at the edge are presented.

Section 2 introduces the research methodology followed to write this survey. Section 3 presents the background of IIoT and edge/fog computing. Section 4 compares the research in this paper with the related literature. Section 5 discusses the security requirements that should be satisfied by IIoT environments. Section 6 presents the attacks that target each layer of the IIoT paradigm reference architecture. Section 7 introduces the state-of-the-art solutions proposed to provide secure deployment of IIoT devices on edge computing. Section 8 presents the opportunities provided by edge/fog computing to IIoT environments, the challenges that IIoT environments face, and the future research directions. Section 9 concludes the survey paper.

## 2. Research Methodology

**Figure 2:**
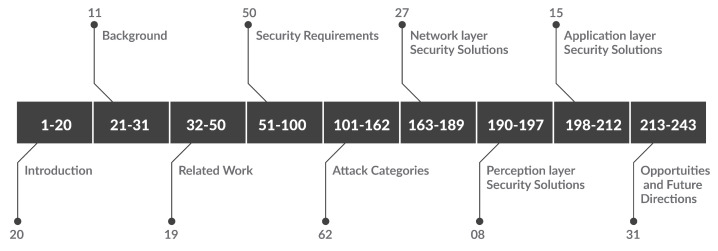
These papers (i.e., 243 articles) were carefully chosen to write this survey paper.

This survey paper utilizes a profound valuation blueprint for an exemplary survey structure. This paper concentrates on the security requirements for IIoT environments, investigates possible attacks targeting these environments, explores security solutions that protect IIoT environments from these attacks, highlights opportunities provided by edge computing, and introduces future directions. We followed a quantitative approach to search for ideas regarding each of these concentrations. However, we focus more on the last six years. The information is collected from various sources, such as journal articles, conference papers, book chapters, and online sources. The collection sources include publication houses and public databases such as ScienceDirect, IEEE Xplore, Springer, MDPI, arXiv (i.e., e-print archive), Hindawi, and ResearchGate. Various keywords related to the topic of the survey paper were employed to search for the state-of-the-art articles in these databases. The publication houses and public databases are queried initially for articles generally related to IIoT security. This provides a recap of the number of papers published in this broad area of research. Consequentially, other general keywords related to edge computing security, the integration of edge computing, and IIoT are queried to give insight into the validity to start writing the survey on this topic. After constructing the survey structure, the search has narrowed to include keywords related to each section, such as IIoT application layer, network layer, perception layer attacks, and AI solutions and solutions that take advantage of edge computing to deal with these attacks. Many articles were returned, but we carefully chose 243 articles to write this survey paper, as shown in Figure 2. Twenty papers were used to write the introduction section. Eleven papers were used to write the IoT/IIoT background and Edge/Fog computing background. Nineteen closely related papers were precisely compared with our survey. The security requirements section was written utilizing fifty papers. Sixty-two papers were employed in the attack categories section. The security solutions section was written using 49 papers, of which 27 papers were utilized to write the network layer security solutions subsection, 8 papers were used to write the perception layer security solutions subsection, and 15 papers were employed to write the application layer security solutions subsection. Finally, thirty-one references were used to write the opportunities and future directions section.

## 3. IoT/IIoT and Edge/Fog Computing Background

This section is divided into two subsections: Section 3.1 presents the concepts of IoT and its IIoT subset, while Section 3.2 introduces two CC extensions (i.e., edge and fog computing).

### 3.1. IoT and IIoT

**Figure 3:**
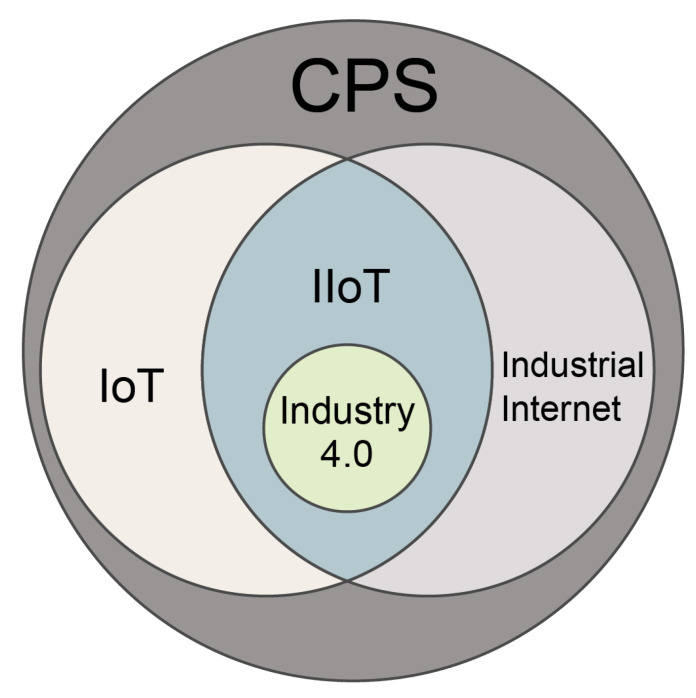
The relationships between CPS, IoT, IIoT, industrial internet, and Industry 4.0.

Figure 3 depicts the relationships existing between the concepts introduced in this subsection. Although IIoT originated from IoT it has different focuses, in terms of practical applications and concepts [21], as shown in Table 1. Namely, the IoT has been designed to improve people’s quality of life and is generally considered consumption-centric. Typical IoT application examples include health monitoring, indoor localization, and smart homes [22]. On the other hand, the IIoT endeavors to enhance the production efficiency of industries (i.e., it is considered a production-centric paradigm). Typical IIoT applications include smart manufacturing, smart transportation, remote maintenance, and intelligent logistics [23]. IoT application system frameworks are generally constructed from scratch, and the utilized sensors are deployed within a small area and are not sensitive to precision [6]. High mobility is one of the main characteristics of IoT devices; the generated data of these devices are of moderate size, and delays can be tolerated to a great extent. Meanwhile, IIoT application system frameworks rely on traditional industrial infrastructures. Thus, the sensors are typically distributed over a large area, and the deployment must be highly precise. Conversely, most IIoT devices are distributed in specific locations; the data generated by these devices are large in size, and only slight delays can be tolerated.

The IoT terminology relates to other famous concepts, such as CPS, Industry 4.0, and industrial internet. The CPS concept, introduced in 2006 by Helen Gill, involves the thorough integration of several technologies, such as sensing and embedded systems (i.e., combining software and hardware), in order to accomplish efficient internal information exchange, resilient real-time feedback, and positive communication between virtual and physical entities [24]. IoT is regarded as a subset of CPS, which assures communication between diverse objects through the internet, depending on unique identifiers. The IoT is supported by the internet, which provides IoT devices with availability, interoperability, universality, and socialization [25]. Another concept, introduced by the IIC and initiated by five U.S. tech companies (i.e., Cisco, Intel, IBM, AT&T, and GE) is industrial internet, which concentrates on data flow enhancement, innovative network standardization, application, construction, and industrial field automated transformation.

Industry 4.0 was introduced in Germany. This global concept utilizes CPS and emerging technologies, such as AI, IoT (i.e., forming the IIoT idea), big data, and CC, in intelligent manufacturers [26]. To recap, CPS connects objects to link the virtual and physical worlds, while IoT utilizes physical addresses in civilian and industrial settings to facilitate communication between objects. The industrial internet uses emerging technologies to depict the prospect of future trends. In this context, industrial internet and IoT are considered subsets of CPS [27,28], and intersect to form the so-called IIoT. Moreover, Industry 4.0 utilizes IIoT, among other emerging technologies, in intelligent manufacturing settings.

### 3.2. Edge and Fog Computing

**Figure 4:**
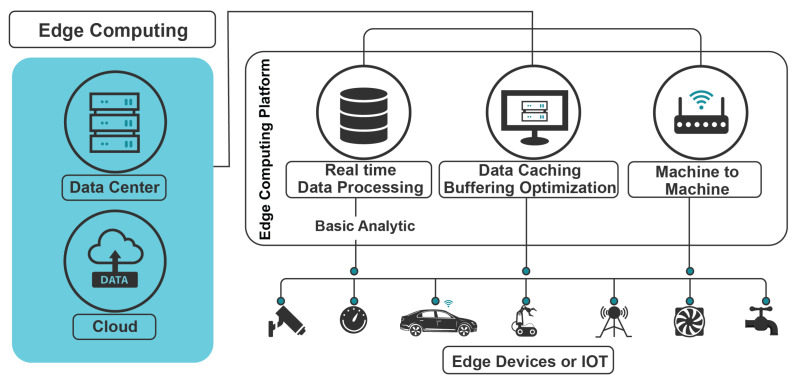
The interaction between edge platforms; the upper layer (cloud servers) and the lower layer (edge devices).

Edge computing is an enabling paradigm that exclusively processes data on the network’s edge. This occurs between centralized cloud servers and end devices (e.g., sensors, actuators, and controllers). One of the main reasons for initiating edge computing is to bring computations closer to hosts, thus reducing delays. Therefore, edge computing enables data to be transferred from end devices to edge computing (i.e., close-to-end devices) and vice versa, instead of imposing that the end devices interact with cloud servers. Thus, as shown in Figure 4, edge platforms can act as clients and servers; namely, clients to cloud servers, and servers to end devices. Acting as servers, they enable end devices to gain the full benefits from edge platforms that can carry out caching, computational offloading, storage capabilities, and processing [29].

Fog computing is another emerging technology that enables edge devices (i.e., end devices and edge platforms) to perform additional computations, handle data, and allocate network resources [30]. Thus, fog computing is not far from the end devices and enables the end devices/edge platforms to carry out most services (e.g., data handling, storage, network resources utilization, and processing) that cloud services can afford [31]. Therefore, edge and fog computing enable delay-sensitive end-device applications to carry out various services in real time. These two emerging technologies have become a viable supplement to CPS and applications in IIoT environments. The following requirements are satisfied by edge and fog computing:

1. System performance enhancement: Data processing can be achieved at the network’s edge, improving the system performance of end devices. Edge platforms can accomplish data processing in milliseconds, reducing the latency and communication bandwidth demand, thus enhancing the system’s performance.
2. Data security and privacy protection: Edge and fog computing can reduce privacy and security risks, as they transmit and store data in decentralized devices (i.e., near-end devices), as opposed to cloud platforms, which provide centralized data protection solutions. Additionally, data leakage at centralized cloud servers affects many end devices, compared to data leakage at edge/fog devices, involving only a limited number of devices (i.e., the end devices nearby that obtain services from edge/fog platforms).
3. Operational cost reduction: When end devices transfer data directly to the cloud, the operational costs related to migrating data, maintaining good bandwidth, and shortening delays are increased. On the other hand, when edge/fog platforms are utilized, the data migration volume, delay, and bandwidth consumption are decreased, leading to reduced operational costs.

## 4. Related Work

In this section, we detail recent survey papers that are closely related to this review, including the state-of-the-art in IoT security, IIoT security, edge computing security, and edge computing in IIoT, as shown in Table 2. Thus, there is a need to survey the secure deployment of edge/fog computing in IIoT environments.

### 4.1. IoT Security Surveys

Meneghello et al. [32] classified IoT attacks into different categories. The authors defined various security requirements for IoT environments, including access control, integrity, privacy, anonymity, authorization, authentication, and resilience, and linked various attacks to suggested security requirements in a well-planned manner. However, this paper lacked a discussion of the role of emerging technologies, such as edge/fog computing and AI, in securing IoT networks. Neshenko et al. [33] conducted a detailed survey and provided a distinctive classification of IoT attacks and vulnerabilities. The authors also broadly discussed research contributions advancing the state-of-the-art; however, these attacks were not linked to security requirements. Famous threats associated with IoT devices discussed by the authors, such as improper patch management, false data injection, and lack of encryption, can be easily linked to security requirements. Furthermore, the authors did not discuss the positive impact of integrating emerging technologies on IoT security.

Kouicem et al. [34] presented an IoT security survey utilizing a top-down approach. The authors explored the security requirements in various IoT application domains, such as smart transportation, healthcare, smart homes, smart grids, and smart cities. Some of the discussed application domains, such as smart transportation, can play a part in the IIoT paradigm. The authors also identified specific security requirements for each application domain. For instance, they defined five security requirements for smart grids: Confidentiality, availability, integrity, privacy, and accountability. In addition, the authors defined some challenges related to IoT devices and networks, such as heterogeneity, privacy, and scalability. It appears that the priority of the security requirements in each application domain differed; for example, confidentiality and privacy are considered more important for healthcare than smart transportation. A significant observation introduced in this survey reveals a security requirement that might be more crucial to IIoT environments, compared to traditional IoT environments, which is related to safety in IIoT environments (e.g., plants). One drawback of this survey is its lack of depth in some sections, such as the challenges part.

### 4.2. IIoT Security Surveys

Lezzi et al. [35] classified IIoT cyberattacks into different categories, while Sisinni et al. [6] investigated the challenges encountered in Industry 4.0 environments, as well as discussing the distinction between operational and information technology in these environments. Neither of the above-mentioned surveys investigated the security requirements in the IIoT paradigm or deeply discussed the effectiveness of adopting emerging technologies to enhance the security of IIoT environments.

Hofer [36] noted the growth of articles focusing on IIoT security and discussed some of these articles. However, the depth of the security discussion was minimal. Hansch et al. [37] defined the security requirements in IIoT open-platform communications, and organized them in a unified architecture format. The authors discussed the security requirements well but did not discuss enough use cases. Other previous reviews [38,39] discussed IIoT security solutions and explored security requirements for the IIoT paradigm. These surveys lacked discussions of the existing security solutions and requirements in depth, however.

Tan and Samsudin [40] introduced a detailed survey of IIoT security, discussed the countermeasures taken by industries to protect their perimeters, discussed current challenges, and suggested future directions for research. The authors categorized the IIoT paradigm into four layers and inspected the countermeasures deployed by industries utilizing the security requirements presented in the CIA+ security certification. One drawback of this survey was a lack of discussion regarding the role of adopting emerging technologies to secure IIoT environments and related use cases.

Serror et al. [41] evaluated emerging IIoT security challenges, including risks, threats, and vulnerabilities. The authors presented a mapping study between security challenges and countermeasures, taking into consideration the distinctive aspects of IIoT deployments, such as continuous connectivity and long-lasting elements. The survey paper is not comprehensive; it complements existing related work.

Jayalaxmi et al. [42] introduced a review of security threats that encounter IIoT environments and the research efforts that protect IIoT environments from these threats. The authors focused on solutions that utilize blockchain technology, machine learning, and deep learning to detect intrusions or mitigate potential threats. Subsequently, a synopsis was presented to outline the pros and cons of each solution. Finally, the authors presented open research problems for the research community and future directions. This review lacks a detailed discussion of the security threats in IIoT environments and sufficient background on blockchain technology, machine learning, and deep learning for readers to understand the foundation of these solutions. Also, the comparison elements that the authors utilize to evaluate existing solutions are shortened.

### 4.3. Edge Computing Security Surveys

Ni et al. [43] presented a complete IoT Edge paradigm and discussed various emerging edge-based IoT applications. The authors investigated mobile edge computing data processing challenges related to privacy, security, and efficiency. Additionally, the authors explored some opportunities, such as secure data de-duplication, secure data aggregation, and secure computational offloading, provided by edge computing that could enhance the computational efficiency of IoT data security. The authors also introduced various motivating future research directions related to data analysis at the edge of networks.

Guan et al. [44] discussed fog computing-related privacy and data security issues. The authors surveyed fog layer security design challenges and also argued that data protection methods used to secure cloud computing are not directly suitable for fog computing. Zhang et al. [45] provided a comprehensive tutorial on edge computing architectures. The authors also discussed edge computing privacy and data security requirements, mechanisms, and challenges. Additionally, the authors suggested various future directions to effectively secure edge computing technology.

### 4.4. Edge Computing in IIoT Surveys

Some reviews have covered the integration of edge computing and IIoT in part. For example, Georgakopoulos et al. [46] presented a roadmap for smart manufacturing to efficiently utilize IoT and edge computing. Seitz et al. [47] introduced two empirical scenarios, in order to demonstrate that IIoT applications can benefit from fog computing. The first practical scenario explains how the fog computing paradigm increases the system availability, compared to cloud computing, and demonstrates how the sensor data can be analyzed in fog computing with a low delay, compared to cloud computing. The second practical scenario is implemented in an industry setting, which indicates a decrease in bandwidth utilization, allowing for the deployment issues associated with cloud computing to be overcome and lead to reliable and efficient IIoT applications. Sitton et al. [48] explained the major state-of-the-art edge computing reference paradigms in Industry 4.0, and compared and contrasted these reference architectures. Steiner and Poledna [49] discussed how fog computing could be integrated with IIoT in an architectural manner, and explored two enabling technologies that can perfectly work with fog computing (i.e., deterministic communication and virtualization). Although these surveys introduced valuable knowledge with respect to integrating IIoT and edge computing, they were not comprehensive.

Other surveys discussed the various main topics related to integrating IIoT and edge computing; however, the depth of these surveys remains low. For instance, Aazam et al. [50] explained how fog computing adds value to IIoT environments, discussed some research challenges, and presented a few use cases; however, they did not discuss the emerging technologies that could enhance the efficiency and secrecy of IIoT environments when integrated with edge computing. Furthermore, the survey paper lacked an in-depth discussion of challenges (security challenges in particular) and sufficient application examples. Basir et al. [8] discussed the Industrial Revolution background and key technologies facilitating industrial transformation. The authors also discussed some challenges related to fog computing. However, their survey was highly concentrated on the communication and network protocols/algorithms used in IIoT, instead of focusing on the challenges associated with adopting edge computing.

Qiu et al. [21] reviewed research articles related to edge computing in IIoT. The authors first discussed the background of edge computing and IIoT in detail. Then, they introduced the edge computing research progress and proposed a prospective edge computing architecture, including technical details such as task scheduling, data storage, routing, security, analytics, and standardization, which could be adopted by IIoT environments. Moreover, the authors discussed the opportunities that edge computing can afford in IIoT environments, such as data security, load balancing, data offloading, and intelligence. They also discussed some challenges concerning the adoption of edge computing into IIoT environments and presented various application scenarios, such as smart grids, smart manufacturing, smart logistics, and ICV. However, the authors only partially discussed security aspects.

### 4.5. Secure IIoT-Edge Deployment

Tange et al. [10] provided a systemic review of IIoT security from 2011 to 2019. The authors concentrated on the security requirements of IIoT and pointed out briefly how fog computing can enhance IIoT security. Although the authors pointed out some security benefits that might be introduced when adopting edge computing in IIoT environments, the depth of the security part when adopting edge computing in IIoT environments was insufficient, and the security challenges and the research progress required to overcome these security challenges were not deeply discussed in this survey. To fill this gap, a survey focused on the security challenges introduced when IIoT environments integrate edge computing, and the research attempts to overcome these security challenges are discussed in this survey paper.

## 5. IIoT Security Requirements

In this section, we introduce the general security requirements that should be satisfied by each communication system, including IIoT environments when deploying IIoT devices for edge computing.

### 5.1. CIA Triad

The famous information security model known as the CIA triad can be regarded as a building block for security requirements or goals. A set of security mechanisms also belong to these three requirements, briefly defined as follows:

- Confidentiality concerns the protection of information in any form. The methods used to satisfy confidentially include access control, encryption, network isolation, and privacy.
- Integrity aims to provide IIoT entities with consistency, authenticity, and accuracy, and allows for building trust with other entities.
- Availability guarantees that the system operates efficiently at all times. Various methods are used to satisfy availability, such as decentralization and redundancy.

Traditionally, the CIA model was utilized in the information security field, implying that this model is exclusively linked to information. Nevertheless, the CIA model is evenly adaptable in other fields, including CPS [51]. Conventionally, industrial environments concentrate primarily on availability, then on integrity, and finally on confidentiality. Meanwhile, with the use of internet-connected devices, this conception should be re-considered, such that all three requirements should be treated equally. Therefore, with the evolution of Industry 4.0 and the IIoT paradigm, integrity and confidentiality must be considered evenly, with respect to availability.

While the CIA security model provides a good foundation and remains of paramount importance when security requirements are specified for a certain system, it is not always valuable for reducing solid requirements back to elements of this security model, if one already has more (e.g., contextual) information that might enable the derivation of a specific security requirement. For instance, we could simply declare that we should keep data confidential at rest; however, such a security goal might not imply the states that a specific confidentiality mechanism should meet. Furthermore, this is open to interpretation. For example, which party should have their information kept confidential? It is difficult to design a constant security goal that works well in all scenarios [10].

### 5.2. Authentication

A major concern in various communication environments, such as IIoT, is authenticating remote entities (e.g., machines, users, and applications) [52]. In the context of IIoT applications, authentication becomes more challenging, due to the nature of IIoT devices, which have limited capabilities due to power constraints, as well as limited storage and processing capacities [53]. Thus, a lightweight authentication mechanism with features such as light computation overhead and minimum transfer size should be designed to overcome these limitations.

Another major concern related to data authenticity is ensuring that data integrity is verifiable and that the data are not altered during transit [54]. Additionally, this applies to configuration files, which should be verified to have been created by authorized entities and not altered since their creation. Considering the nature of IIoT devices, IIoT environments require authentication solutions that satisfy the trade-off between lightweight and secrecy, as well-known authentication mechanisms will not be able to be adopted in such environments [55].

In IIoT environments that utilize edge computing, blockchain-based authentication is a suitable preference to authenticate remote entities and provide data integrity. A representative model is the one proposed by Wang et al. [56]. The authors proposed a lightweight message authentication framework based on blockchain that ensures message security while utilizing minimal computational overhead for IIoT resource-constrained devices. This framework utilizes edge servers to oblige IIoT devices to perform cross-domain authentication and effectively decrease redundant communications between these devices. The secrecy of this model is analyzed using a random oracle scheme, proving its resistance to several attacks.

### 5.3. Access Control and Authorization

Access control is significant in various circumstances. Devices in IIoT environments should be given permission to access edge network resources based on their privileges. For instance, system administrators would be given more permissions (e.g., for updates deployment) than normal users. In some situations, IIoT devices run on two operation modes: an administrator and a normal user. In this context, adequately separating privileges is one of the biggest challenges that systems (e.g., SCADA) in IIoT environments encounter [57]. On the other hand, unauthorized users should be prevented from accessing sensitive data or altering data [58].

Access control is usually regarded as authentication-reliant, as it is necessary to authenticate users before enforcing the access policy. Thus, access control mechanisms are typically similar to authentication mechanisms. Access control can consume IIoT device resources, as the IIoT devices must interact with authorization servers on the edge before accessing certain resources. Access control is somehow affected by availability, especially in the IIoT environment (i.e., it is highly distributed); thus, access control policies should always be available to IIoT devices.

The access control function acts as an agent between a specific user or one of the IIoT devices processes and system or edge network resources, including operating systems, firewalls, routers, applications, and databases. If a specific party (i.e., a user or a process) wants to access a resource, this party must be authenticated first. The authentication mechanism decides whether the party as a whole is allowed to gain access to the system or not. Subsequently, the specific request initiated by the party is permitted or not by the access control function. A network administrator or security personnel typically creates and sustains an authorization database containing information determining the access type permitted to that party. The access control mechanism confers the authorization database to decide whether to permit access to this party [59].

An example is the access control and authorization mechanism developed by the Kantara initiative, known as UMA [60]. UMA is a capability-based scheme that enables a particular entity possessing a capability and an access token to access a given resource. UMA is a suitable fit for IIoT environments because it is a user-oriented standard. In typical UMA architectures, the resource (e.g., a specific file) owner stores the resources at a resource server and controls the resources. A designated authorization server should be incorporated to protect the resource server. The resources must be registered to the authorization server by the register server and given appropriate policies for users or processes. The user or process should obtain an authorization grant by sending a request to the resource server. To issue a ticket to the process, permission must be registered by the resource server on the authorization server. Then, the process reveals the ticket to the authorization server to grant permission. If the authorization server gives the permission, an RPT is issued to the process. The process can access the requested resource using the RPT [61].

The conventional IIoT environments use TTP as middleware to authenticate devices before enforcing access control policies. Since TTP is involved, data privacy preservation is a big issue. Additionally, other issues appear due to the involvement of TTP such as SPOF, trust, and vulnerabilities. Therefore, IIoT devices must be collectively and collaboratively authenticated in a decentralized manner using for example blockchain technology. For example, Dwivedi et al. [62] introduced a solution to these problems utilizing blockchain technology. The authors present a fully decentralized system for IIoT devices that does not rely on TTP using IPFS Ethereum smart contracts. The proposed framework also consists of data accessing policies designated for end users.

### 5.4. Resilience and Maintainability

Resilience is defined by ICS in its IIoT security framework as an emerging mechanism equipped with a system that normally carries out the assigned missions, even if it encounters adversarial conditions. This system must avoid, absorb, and coordinate dynamically to work properly and complete its designated tasks. Once the system is infected, it should be capable of reconstituting its operational capabilities. This terminology is similar in concept to other security terminologies, such as reliability, safety, and trustworthiness [63]. Resilience is one of the most significant security challenges in IIoT environments [64].

IIoT networks should provide some mechanisms to guarantee that operations on IIoT systems will be normally performed, even if a part of the system is compromised. This could be done in IIoT networks by forwarding some of the current tasks from the infected part to another part of the system, or even to a different system. In the research community, this technique is usually called diversity, redundancy, or hardening [65]. This concept is applicable in WSNs, in which a sufficient number of sensors are deployed to ensure redundancy. Such a scenario aims to isolate compromised sensors when infection occurs, re-routing the new measurements to the other sensors in the network until the issue is resolved.

Maintainability can be described as the capacity to configure, reconfigure, and update a system or part of a system. This security requirement is crucial in the IIoT paradigm, as the software in IIoT devices must have the capacity to be updated to be protected against previously unknown cyberattacks [66]. Updating software is considered a valuable countermeasure against various threats, as it helps to continuously modify firewall configurations at the network’s edge once the IDS detects new threats. Additionally, software vulnerabilities can be restored by utilizing software patches in routine software updates.

Various IIoT devices communicate with each other and other traditional devices through the internet, which is insecure by design. Therefore, regularly updating IIoT devices and patching their vulnerabilities is essential to maintain their resilience against cyberattacks. Some proposals, such as the one proposed by [67], utilize blockchain technology to provide secure and reliable updates for IIoT devices. The authors employ an incentive protocol in which a specific agent supplies the updates and utilizes a smart contract to create a pledge to give collaborated nodes that transmit the updates to the IIoT devices financial incentive. To obtain the financial incentive, the collaborated nodes should provide the agent with proof-of-delivery. The collaborated nodes should employ DAPS via an attribute-based signature to perform the fair exchange and obtain the proof-of-delivery.

### 5.5. Privacy

Privacy is a significant security requirement for individuals, companies, and governments. Due to the emerging demand for cloud storage services, privacy preservation has become a critical issue [68]. Modern devices generate variable amounts of data, making users susceptible to privacy violations, in which detailed profiles can be created for users from the generated data without their permission [69]. Additionally, applications can violate privacy by revealing personal information about a user’s habits, movements, and interactions with other users [70]; for instance, a user’s location could be tracked by one of the applications they install on their devices.

Moreover, some websites (e-commerce websites, in particular) collect information about users, such as previous visits to products, shopping carts, and even credit card information. The collected information might be released to other companies without the user’s permission. Another challenge is data capturing in transit, which may reveal personal information about people and objects.

Data redundancy in IIoT environments can be resolved through mechanisms that store data at rest. However, privacy protection and data security are two main challenges for stored data. Stored data can be encrypted and retrieved without violating users’ privacy using some encryption methods, such as attribute-based searchable encryption. For example, Niu et al. [71] proposed an attribute-based searchable technique that incorporates an online and offline encryption scheme that relies on a reusable ciphertext pool capable of reducing the computation burden and outsourcing decryption mechanism that utilizes an edge server capable of decreasing the overload on the resource-constrained devices via outsourcing decryption. Additionally, the server can be authenticated by a specified authentication server in the searchable location.

### 5.6. Security Monitoring

Dynamic security monitoring of systems behavior is provided by famous tools known as IDSs. These tools can detect threats targeting networks and provide the required response procedure. It is substantial for any network—including IIoT environments—to monitor communications, identify threats, and respond to known and unknown intrusions if needed [72,73,74]. One reason underlying the importance of IDSs is that old and less-secure devices (i.e., those that are difficult to patch to deal with known vulnerabilities) could connect to the network, which demands continuous security monitoring [75]. These devices might become a target of a DDoS attack. Then, they may become part of a botnet that can launch attacks against other legitimate IIoT devices in the network.

Capturing and investigating exchanged data, networks, and services using passive network traffic monitoring and analysis systems are of paramount significance in coordinating networks and identifying security issues in a timely manner [76,77,78]. The IDS can be identified as a tool that monitors network traffic to detect attacks compromising the CIA model of a given information system [79].

An IDS can be operated in three phases. The first phase is responsible for monitoring traffic or data, which depends on host- or network-based sensors. The second phase is responsible for analyzing the captured network traffic or collected data. This phase utilizes feature extraction or pattern identification techniques to accomplish the task. The third phase involves detecting threats using two well-known approaches: misuse detection and anomaly detection [80].

Misuse-based intrusion detection methods gather known signatures and patterns of familiar threats in a database and compare incoming traffic with the database entries to detect attacks [81]. Misuse-based intrusion detection techniques have disadvantages, such as the high cost of signature matching, increased number of false alerts, and overload of network datagrams [82]. Additionally, the memory constraints of IIoT devices make it difficult to implement misuse-based IDS in those devices, due to the burden of a large number of signature entries in a database [83]. Moreover, the databases assigned for attack signatures and patterns must be periodically updated. Misuse-based IDSs require previous knowledge to be able to identify suspicious activities. Thus, unknown attacks may not be detected by this type of IDS [84].

Anomaly-based IDS methods maintain the situation in which genuine devices generate normal data in the network and assess monitored data accordingly to identify anomalies (i.e., outliers that deviate from the normal data) [85]. These outliers are usually generated from noise or other incidents, which could result from utilizing a hacking tool. Therefore, unusual activities resulting from the existence of attackers would leave footprints in the infected network [86]. Therefore, attacks (including unknown ones) can be detected by anomaly-based IDSs using these footprints. In short, an anomaly-based IDS method creates a pattern of normal data generated by legitimate devices in the network, updates the pattern periodically, monitors network traffic in real time, and compares the monitored traffic with the normal pattern; if any deviation from the normal pattern exists, it may indicate an intruder [87].

The open connectivity nature and the widespread use of IIoT devices make them susceptible to cyberattacks. Additionally, the prevalence and heterogeneity of IIoT devices increase the difficulty of presenting a centralized cyberattack detection method. Thus, proposing decentralized approaches in close proximity to IIoT environments to detect cyberattacks is vital. For instance, Javeed et al. [88] presented an edge-based mechanism capable of securing communication in IIoT environments. The proposed approach utilizes AI to detect cyberattacks. The authors combine two well-known RNN architectures: GRU AND BiLSTM to distinguish the anomalies from normal traffic and edge computing to facilitate the routing flexibility and interoperability of the heterogeneous IIoT devices.

### 5.7. Secure Data Sharing

Securing data are important in digital paradigms, including IIoT. Various research papers have described data confidentiality as a substantial security requirement [89,90,91,92]. However, integrity and availability have a quantifiable economic effect and, hence, are considered more important than data confidentiality in traditional industrial settings [93,94]. Due to the evolution of the ICS as an integral part of the IoT paradigm, data confidentiality significance has become intelligible, due to the interactions in the ICS between devices and users that generate private data.

Companies consider secure data sharing an important security requirement to integrate Industry 4.0 [95,96]. Additionally, some companies have hesitated to employ data sharing-based approaches, such as smart maintenance, fault detection and prevention, and cloud services, as they believe that the data exchanged from their facilities to service providers might not be sufficiently protected [97]. Other researchers have supported the sentiment that organizations are hesitant to deploy cloud services or depend on cloud providers to supply data storage and sharing to customers [98]. Another serious challenge occurs when data breaches occur internally.

There exist various other challenges related to IIoT devices, applications, and environments. Data security techniques must be light to be equipped in IIoT devices with limited resources. Additionally, these techniques should be able to operate on heterogeneous devices. Some critical IIoT applications demand a full-fledged data security mechanism, so, it is infeasible to implement the mechanism on a resource-constrained device (i.e., an edge node is favorable). Moreover, data security is important as, in industrial settings, it is important to share data to enable various intelligent capabilities; therefore, the data are usually sensitive [99]. An important characteristic of Industry 4.0 is the utilization of available data in an intelligent and efficient manner. Thus, the ability to share data with other entities in an Industry 4.0 environment or outside the environment’s boundaries is significant to fulfill this requirement.

The IIoT devices generate large amounts of data; the generated data must be processed via distributed computing and stored in one or more nodes for analysis and retrieval. This presents security and privacy issues and introduces scalability challenges. To protect confidential data and share data securely, efficient protocols are required, such as the one introduced by Hosen et al. [100], a secure peer-to-peer and group communication framework that utilizes edge computing to support IIoT systems’ secure data sharing. The framework uses consortium blockchain, IPFS-based immutable data storage mechanism, and a detection scheme to protect confidential data at rest and in transit and detect cyber threats. The authors improved the PoV consensus algorithm to reduce the latency during block mining in blockchain technology because of the overhead and errors that cause POF. The detection scheme is based on two deep learning algorithms known as autoencoder and RNN; the former is used to reduce the dimensionality of the generated day, and the latter is used to classify traffic as legitimate or an attack. After detecting the attack, the model can identify its type.

## 6. IIoT Attack Categories

**Figure 5:**
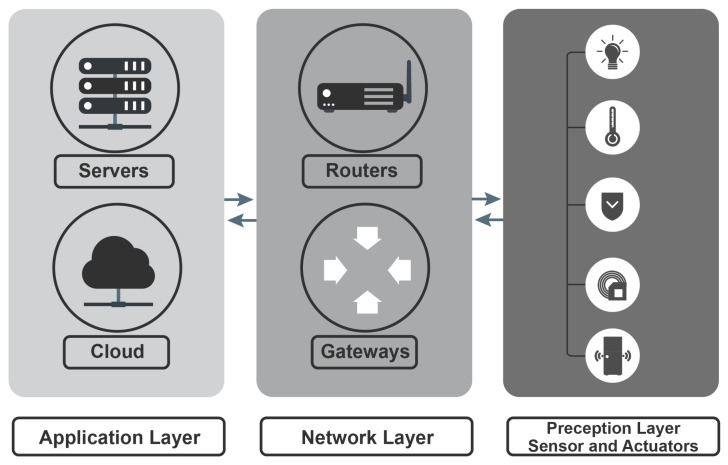
Framework of the three traditional IIoT layers: perception, network, and application.

IIoT environments comprise various devices, ranging from tiny embedded systems to full-fledged servers. Thus, it is significant to highlight the security challenges at different IIoT layers. As shown in Figure 5, the traditional IIoT layer architecture consists of three layers: The perception layer, the network layer, and the application layer. Each layer has its own enabling technologies and unique features. Thus, this section discusses these three layers and the challenges IIoT applications encounter when applying security requirements in industrial environments [101]. Table 3 depicts popular attacks that target the three IIoT layers, along with their common countermeasures.

### 6.1. Perception Layer Attacks

The perception layer (a.k.a. device layer) consists of devices equipped with various objects, such as sensors, cameras, robots, and smart meters, as shown in Figure 5. This layer is responsible for identifying and gathering information related to the target sensor. This information comprises measurements of quantities such as movement, chemicals in a specific environment, vibrations, heat, acceleration, or humidity. The collected data are transmitted to the lower layer (i.e., network layer), and eventually conveyed using guided (e.g., industrial Ethernet cable) or unguided media (e.g., WiFi) to an information processing system at the edge [124]. This subsection concentrates on attacks targeting the perception layer.

#### 6.1.1. Node Capture Attacks

In this kind of attack, the attacker can physically obtain or replace an IIoT node or modify certain hardware. This type of malicious act leads to exposing sensitive information related to digital rights coordination, such as cryptography keys or access keys. Once the attacker gains access to the IIoT device, they can then malignantly act to harm other devices in the network [125].

#### 6.1.2. Jamming Attacks

This type of attack can disrupt or alert communication of IIoT devices by tampering or interfering with the access mode of wireless communication. Thus, IIoT devices will be prevented from transmitting data to other network entities successfully [126]. Attackers can jam the wireless signal remotely utilizing a powerful passive transmitter. They can also use shielding techniques to avoid defensive mechanisms. Radio noise that matches the frequency of a specific system can be utilized to interfere maliciously with RFID systems.

#### 6.1.3. Sleep Deprivation Attacks

This family of attacks prevents IIoT devices from resetting to sleeping mode by inserting infinitely looping codes into the device’s memory or making hardware modifications. By default, IIoT devices are battery-constrained and remain on sleep mode when they do not transmit or receive information, to preserve battery; however, these attacks can drain the batteries of IIoT devices by actively waking them up, eventually shutting them down completely (this is a type of DoS attack) [127].

#### 6.1.4. Replay Attacks

Without authentication mechanisms, an intruder can capture a previously legitimate message transmitted from an IIoT device to another entity, then modify and replay the message to its final destination [128]. This kind of attack is possible when the authentication is applied in a certain IIoT environment. The intruder can eavesdrop on the wireless channel, capture the message, clone, and use the authentication code in the captured message (i.e., generated by the sender).

### 6.2. Network Layer Attacks

As shown in Figure 5, once the data are handed from the perception layer to the network layer, the network layer identifies the path the message takes to reach the receiver (this path includes the first edge router, which is responsible for forwarding the message to the next router on the route) [129]. The network layer aims to transfer network packets (a.k.a. datagrams) between heterogeneous networks transmitted by various IIoT devices. These network packets are sent by the IIoT device interface using a communication protocol, passing through various communication links [130]. The transmitted packets from IIoT devices are usually obtained by nodes at the edge, such as routers or gateways for processing and forwarding to the outside world. Therefore, IIoT devices and edge nodes are susceptible to network layer attacks. This subsection introduces the attacks that target the network layer.

#### 6.2.1. Eavesdropping Attacks

This type of attack enables the intruder to listen to the ongoing exchange of messages between IIoT devices in the communication channel. The message exchange can include sensitive information, including passwords and bank information in plaintext if encryption is not applied [131].

#### 6.2.2. Sybil and ID Cloning Attacks

A Sybil attack occurs when an intruder steals the identity of a legitimate IIoT device to disturb the communication between devices. Additionally, an intruder can maliciously possess various identities to deceive IIoT devices into believing many IIoT devices are in the network [132]. On the other hand, a clone ID attack can be defined as spoofing a legitimate node’s identity and pretending that the attacker has the identity of another legitimate node in the network. An attacker can launch this attack to access more devices in the network [133].

#### 6.2.3. Wormhole Attacks

This type of attack allows two attackers to create a virtual long-distance tunnel, which is created to force the other devices in the network to transmit their packets through that tunnel [134,135]. Additionally, the exchanged information could pass through the intermediate legitimate nodes to drain their batteries [136].

#### 6.2.4. Denial of Service (DoS) Attacks

An intruder can launch this type of attack to sabotage bandwidth or network resources, which can be accomplished by actively transmitting a large number of packets to the devices/servers connected to the network indefinitely or temporarily to make them busy and eventually prevent them from doing their usual activities. This attack might also drain the batteries of IIoT devices, leading them to completely shut down [137,138]. Another subset of DoS attacks is DDoS, which compromises normal IIoT devices that do not have appropriate security protection to become a source of attack traffic. This attack can be categorized into logical and flooding [139]. A logical attack allows the intruder to transmit deceiving messages to misguide normal users into believing that the service’s application or service on the machine they are contacting is unavailable (i.e., fully occupied). A flooding attack targets edge IIoT devices or servers by transmitting numerous amount of packets, making the target devices unable to process these packets and eventually become unavailable to normal users in the network (i.e., they cannot reply to normal requests from legitimate users) [140,141].

Edge computing is more susceptible to DoS attacks than cloud computing, as services are provided by edge IIoT devices, which cannot be equipped with suitable defense mechanisms due to computational limitations. Additionally, attackers target edge devices and use them as sources to launch attacks on nearby edge servers; hence, the attacks may be more severe, compared to when targeting far-away cloud servers (in which case, the traffic would pass through various routers and might be blocked before it reaches the cloud server). In this regard, a memorable case is when 65,000 IoT devices were targeted and exploited to launch malicious packets against famous services, such as Dyn (a company that offers services to control, coordinate, and optimize online infrastructure), Kerbs (a daily blog that covers cyberattacks), and OVH (a giant European hosting provider); this attack is known as the Mirai botnet [142]. The DoS attacks have various subtypes including:

1. A selective-forwarding attack is a type of DoS attack. In this attack, the attacker may choose to forward certain packets (e.g., RPL control messages) and drop the rest of the packets to disrupt the route [143]. This attack can have more severe consequences when combined with other attacks, such as sinkhole attacks.
2. The intruder launches this attack to lure network entities to believe that it is the sink node (i.e., a node in a network with stronger capabilities than other nodes in the network), to forward network traffic to it. The forwarded traffic is eventually transmitted to the attacker, and might not reach the intended receiver [144].
3. This attack can be launched by a malicious node that acts as a hole (a node that forces the other network entities to route the packets to it and drop the forwarded packets), to degrade IIoT network performance [145].

#### 6.2.5. Man in the Middle Attacks

The attacker can launch this attack to become a “man in the middle” of ongoing communication between two legitimate IIoT nodes. The attacker can then monitor the communication in real-time, as well as intercept and alter the exchanged messages [146].

### 6.3. Application Layer Attacks

As shown in Figure 5, the last layer in the traditional IIoT layer architecture is the application layer. The application layer presents data and provides IIoT users with various applications, such as smart transportation, smart manufacturing, and intelligent logistics [147]. Before we dig into the application layer attacks and for illustration purposes, we will present an example of application layer attacks (i.e., ransomware attacks) that might target IIoT environments. Recently, IIoT devices have become tempting targets for applications layer attacks. IIoT devices and analytics systems deliver various benefits for industries that help increase growth rates and market capitalization. However, any chance of sudden downtime would cause substantial losses that could cost the industry up to USD 260,000 per hour. To that extent, attackers find IIoT devices the ideal targets to gain profit because if the attacks launched by the attackers succeed, the industry will likely pay the ransom to avoid the downtime that might occur due to these attacks. IIoT devices are susceptible to ransomware attacks in some IIoT deployments, such as Brownfield-IIoT deployments. In these deployments, the legacy systems deployed at the edge should be connected to the internet. These systems are vulnerable to several security issues because these systems usually do not support decent security countermeasures such as encryption, updating, and patching. The workstations at the edge that employ these systems (particularly ones that use unpatched operating systems) are the medium attackers could exploit to spread their attacks in the IIoT environment because they have direct or indirect connections with ICSs. The ICSs usually use workstation interfaces through the OLE protocol for process control.

Additionally, other processes, such as NetBIOS and SMB, are utilized by ICSs for implementation and configuration purposes. Once a ransomware attack infects the workstation via and malicious USB, malicious link, or suspicious file, the ransomware can be easily distributed to critical ICSs. In the last few years, a ransomware version known as “WannaCry” exploited workstation interfaces to spread attacks in IIoT environments, affecting several industries worldwide [148]. The application layer is susceptible to various security issues, listed as follows:

#### 6.3.1. Malicious Code Injection Attacks

Attackers can exploit the vulnerabilities associated with the debug modules to inject malicious codes. Once the attackers inject the malicious codes, the attacker can then perform unwanted activities on the affected device [149]. Additionally, the attacker may be able to carry out malicious activities on the entire network through the affected device. Additionally, IIoT devices can be infected when they upgrade their firmware/software using an OTA utility. More specifically, the attacker can inject a virus into the IIoT device when the device is installing a scheduled firmware update; hence, this action demands rebooting the IIoT device to be effective. To protect IIoT devices from such attacks, there should be a suitable authentication mechanism and identification for the edge devices, as well as ensuring that the updates and upgrades that can be installed on IIoT devices are trustworthy (i.e., do not carry malware).

#### 6.3.2. Cross-Site or Malicious Scripts Attacks

These vulnerabilities can be exploited by malicious nodes, through websites visited by IIoT users. Particularly, suspicious websites could be equipped with malicious scripts that decoy the user’s system to become infected, thus revealing the user’s data [150]. Such malicious scripts can be created using any scripting language, such as JavaScript, like any other legitimate script, and run by any internet browser. One possible threat of cross-site and malicious scripts is their ability to lure users to upload data, even without verification [15].

#### 6.3.3. Malware Injection Attacks

In this type of attack, the intruder targets a victim edge device’s service requests to inject malware into that device’s system or the network [151]. This attack leads to disruptive threats to system security and data integrity. Both edge servers and devices are susceptible to this kind of attack. The edge server can be targeted by a malware injection attack known as SSI. This attack can be categorized into four classes: XML injection, CSRF injection, XSS injection, and SSRF injection. Edge devices are prone to a malware injection attack known as DSI (e.g., RCE or reaper), in which the attacker injects malicious code into the targeted IIoT device [152,153].

#### 6.3.4. Data Distortion Attacks

In this type of attack, intruders eavesdrop on the wireless channel, intercept the packets transmitted between network entities, distort them, and forward them to the receiver [154].

#### 6.3.5. SQL Injection Attacks

This type of attack exploits the vulnerabilities of applications that retrieve and transmit information from and to the databases. This family of attacks can also modify the running SQL query by maliciously launching a query fragment; for example, through a web form. Consequentially, the attacker can gain access to the database and alter the database schemes, tables, tuples, or attributes [155].

#### 6.3.6. Ransomware Attacks

A ransomware attack is a subset of the malware attacks family, where the attacker hijacks IIoT devices or files and asks for compensation (usually money) to restore access to IIoT devices or decrypt files, such that the victim device can use them again. The cyber-criminals who launch this type of attack usually interact with the victims and ask them to pay a ransom (e.g., Bitcoin) in exchange for decrypting the files or regaining access to the IoT devices [156].

#### 6.3.7. Side-Channel Attacks

This type of attack utilizes publicly available data (i.e., insensitive data) to deduce confidential data by relating them with the user’s private data. The attacker takes advantage of publicly available data on the edge computing infrastructure and feeds them as input to ML, DL, or anonymous algorithms to generate the desired output (e.g., sensitive information). Side-channel attacks may target any network entity, and intruders can utilize various methods to launch side-channel attacks, such as timing attacks, cache attacks, and electromagnetic attacks [157,158,159].

#### 6.3.8. Authorization and Authentication Attacks

In these types of attacks, the intruder utilizes fake credentials to gain access to protected resources. Ordinarily, edge servers and devices are authenticated in edge computing, to authorize edge devices to gain access to the services or resources placed on the edge servers. These types of attacks can be classified into four groups: Threats that exploit authentication methods, threats that target authorization protocols, dictionary attacks, and over-privileged attacks [160].

In a dictionary attack, the attacker creates a file of the most-used passwords and tries every possible password in a matter of minutes, to determine the correct credentials that allow the attacker to gain access to the resources of a specific user [161]. In authentication and authorization protocol attacks, the adversary exploits authorization or authentication vulnerabilities to reveal the authenticated user’s credentials, thus gaining access to the resources or services at the edge servers as an authorized user. In over-privilege attacks, the intruder can shut down or gain access to the system as an authorized user by inserting malware. This attack can be launched in various forms, such as changing a smart home door pin and retrieving and utilizing the user’s voice records [162].

## 7. State-of-the-Art IIoT Secure Deployment on Edge Computing

Various IIoT edge computing entities utilize communication protocols, sensing capabilities, and data processing techniques to interact with each other, accomplishing various advances in many applications. Edge computing plays an integral role in enhancing the performance of the IIoT paradigm. For example, low latency has become one of the main characteristics distinguishing edge computing from cloud computing, thus enhancing the performance of real-time applications. Additionally, edge computing improves the security of IIoT environments, to some extent. However, traditional security mechanisms cannot be directly applied to edge computing and completely satisfy the security requirements discussed in Section 5, as it is difficult to predict security risks when designing the security model. Furthermore, security threats related to networks, data, or applications emerge as technologies are integrated with each other (i.e., adopting edge computing to IIoT environments will bring more threats to the IIoT paradigm related to edge computing). Some well-known security risks related to the integration of IIoT and edge computing, as well as state-of-the-art solutions aimed at these risks, are discussed in this section.

### 7.1. Network Layer Security

An attack launched from the edge network could threaten all of the edge functional entities and may propagate to the whole communication network (e.g., eavesdropping on the communication link or injecting malicious traffic to the broadcast address in the network) [163]. Intrusion detection and prevention are two important research interests proposed to protect edge network security in IIoT environments. Many current solutions to combat IIoT network layer attacks rely on emerging technologies, such as AI- and Blockchain-based solutions, to provide the necessary detection and prevention mechanisms, as detailed in Table 4. For example, Diro and Chilamkurti [164] utilized the LSTM algorithm to detect attacks on distributed fog environments that might target IIoT devices. This technique is the first step to improving the security of fog computing, by accurately and precisely detecting various attacks that might degrade the network performance and malfunctioning network entities. The authors validated the proposed technique using two datasets—ISCX (Found at https://www.unb.ca/cic/datasets/ids.html (accessed on 26 January 2023)) and AWID (Found at https://icsdweb.aegean.gr/awid/ (accessed on 28 January 2023))—and compared the proposed method with LR. The technique yielded a promising accuracy of 98.22% on the AWID dataset and 99.91% on the ISCX dataset. The proposed technique was better than LR by 9% on the ISCX dataset; however, it took a significantly longer time to train the proposed method, compared to LR.

Chekired et al. [165] proposed a distributed and hierarchical intrusion detection system to detect attacks targeting the fog architecture. The proposed solution was mainly designed to detect false data injection attacks that target smart meters in the power grid. The proposed technique consists of three layers: AMI, fog, and cloud. Each layer incorporates various IDSs that hierarchically detect intrusions in a cooperative manner. The fog layer assimilates three types of IDS: Fog IDS, residual area network IDS, and HAN IDS. The authors then adopted a stochastic MC to differentiate malicious activities from normal traffic. The authors demonstrated the effectiveness of the proposed technique using real electricity data generated from Toronto.

Huang et al. [166] presented a defense approach to prevent DDoS attacks in IIoT environments. The proposed technique relies on a multi-point collaborative capability, deployed at the edge to detect DDoS attacks and protect IIoT devices from adversaries. The collaborative defense aspect of the proposed technique is accomplished through the use of blockchain technology, which is adopted to securely distribute defense information throughout the IIoT environment. Additionally, the authors introduced a swift defense information distribution technique, to minimize the information sharing latency and enable the proposed method to respond promptly. The authors also employed two deep learning-based mechanisms to differentiate normal traffic from attacks using an LSTM-Attention network, the attack traffic was further categorized, and the attacks were detected using a 1D CNN architecture. Furthermore, the authors used the classified attack feature representations to acquire new feature information and, hence, produce defense information and improve the robustness of the security system. The classification part based on deep learning was evaluated and compared with baseline models (i.e., SVM, MLP, and *k*NN). The deep learning-based techniques obtained superior results, compared to the baseline models, in terms of precision, recall, F1 score, and accuracy. Experiments conducted on the DoS2019 dataset (Found at https://www.unb.ca/cic/datasets/ddos-2019.html (accessed on 9 February 2023)) also demonstrated that the swift sharing approach could decrease the propagation delay when distributing the information, thus enhancing the response time and better protecting the devices from DDoS attacks. The proposed LSTM-based approach achieved high performance in three performance metrics (i.e., 99% precision, 98.7% recall, and 98.8% F1 score), while the 1D CNN-based method achieved slightly better results than the LSTM-based approach (i.e., 99.3% precision, 98.9% recall, and 99.1% F1 score).

Mudassir et al. [167] presented three accurate deep learning-based approaches capable of detecting botnet attacks that target the IIoT environment. The three techniques are based on ANN, RNN-LSTM, and RNN-GRU, respectively, and were evaluated on the BotIoT dataset. The ANN-based approach achieved the highest performance, in terms of accuracy (99%), although the other techniques obtained similar accuracies (98%). However, the RNN-GRU-based techniques performed slightly better in terms of detecting attacks with minimum samples, such as DoS and DDoS targeting HTTP protocol. The performances of the three models, in terms of precision and recall, were not high, particularly in classifying attacks with a small number of samples. Thus, the authors improved their performance by under-sampling the majority class to create a balanced dataset. The proposed methods achieved better results, in terms of precision and recall, on the balanced dataset. However, deploying such techniques on IIoT networks may pose an issue, considering the constraints of the devices, as the deployment of deep learning-based approaches typically requires high computation and memory usage.

Tsogbaatar et al. [168] introduced a framework using an ensemble of deep learning models as a building block to detect IoT threats utilizing SDN. The proposed framework consists of three modules: An anomaly detector module, device status prediction, and smart flow management. Stacked deep auto-encoders are used to extract features and feed them into the ensemble deep learning model. The proposed system was evaluated on the N-BaIoT and costumed datasets, and accomplished superior results on even a 1% imbalanced dataset, compared to related works, achieving an improvement of approximately 3% over a single deep learning model.

Popoola et al. [169] proposed using dimensionality reduction and intrusion detection techniques to identify threats in IoT environments. The dimensionality reduction part of the framework was based on LAE, while the intrusion detection part was based on B-LSTM. The authors analyzed the long-term inter-related changes using B-LSTM after the LAE had reduced the feature set to accurately identify network traffic samples. The proposed framework was validated on the BotIoT dataset, yielding promising results. The conducted experiments demonstrated that the utilized feature reduction technique remarkably improved the memory space, by approximately 92%, and performed better than state-of-the-art dimensionality techniques by up to 27%. The performance of the proposed framework, in terms of MCC, was high; obtaining 93.17% in binary classification scenarios and 97.29% in multi-class classification scenarios.

Popoola et al. [170] introduced a botnet detection technique based on deep learning which is capable of dealing with imbalanced network traffic data. The authors adopted the SMOTE algorithm, which produces additional samples for classes with a small number of samples, to attain class balance. Consequentially, the authors fed the balanced data into a deep RNN to acquire knowledge of the hierarchical feature representations and, thus, distinguished attacks from normal traffic. The authors conducted two types of experiments using the BotIoT dataset: Without and with the SMOTE algorithm. The first experiment proved that the imbalanced data affected the results (in terms of recall, precision, F1 score, AUC, GM, and MCC). On the contrary, the SMOTE-RNN-based approach yielded superior detection results, compared to state-of-the-art models, achieving 99.75% recall, 99.50% precision, 99.62% F1 score, 99.87% AUC, 99.74% GM, and 99.62% MCC. The proposed solution utilized the characteristic of RNNs, in terms of distinguishing samples in historical time-series data, which have achieved high accuracy in many fields, including intrusion detection systems. However, the time required to detect intrusions is not negligible, which is a key issue, as this technique must be deployed on resource-constrained edge devices.

Jayalaxmi et al. [171] proposed a botnet detection technique based on deep learning to protect IIoT networks. This method adopts a CFBPNN architecture and a feature selection method known as CFS, in order to minimize the time required for the intrusions and improve the detection rate performance. Additionally, the authors utilized a time-series technique known as NARX to examine the elements that have a high impact on the target class, to anticipate the behavioral pattern. The authors conducted various experiments on five datasets to evaluate their proposed framework; namely, NF-UNSW-NB15, NF-CSE-CIC-IDS2018, NF-ToN-IoT, NF-BoT-IoT (these four datasets can be found at https://staff.itee.uq.edu.au/marius/NIDS_datasets/ (accessed on 12 February 2023)), and ToN-IoT-Windows (this dataset can be found at https://research.unsw.edu.au/projects/toniot-datasets (accessed on 20 February 2023)). The authors compared the proposed framework with various neural network models; the results indicated perfect accuracy, an outstanding F1 score, and good precision of the proposed model.

Alani et al. [172] proposed an effective botnet detection method using packet inspection and machine learning. The proposed framework also utilizes a feature selection technique to reduce the feature set and the detection time. The feature selection method chooses only seven important features, extracted from the network packet fields. These features are fed into the machine learning algorithm, in order to train it. The proposed detection technique and feature selection capability achieved higher than 99% accuracy.

Popoola et al. [173] introduced an FDL-based technique to detect zero-day botnet attacks and protect IoT edge devices from data privacy leakage. The authors presented an optimal DNN architecture to classify the captured network traffic. The models of the DNN architecture are independently trained in multiple IoT edge devices, remotely managed by a model parameter server, and local model updates are aggregated using the federated averaging algorithm. Various messages exchanged between IoT edge devices and model parameter servers were used to generate the global DNN model. The authors utilized two datasets to validate their proposed framework: BotIoT (found at https://research.unsw.edu.au/projects/bot-iot-dataset (accessed on 2 March 2023) or https://ieee-dataport.org/documents/bot-iot-dataset (accessed on 2 March 2023)) and N-BaIoT (found at https://www.kaggle.com/datasets/mkashifn/nBaIoT-dataset (accessed on 2 March 2023)). The proposed framework presented a high performance in classification metrics and can ensure data confidentiality and privacy. As the training data are distributed between edge IoT devices, the required memory space and storage are minimal for each IoT device. Additionally, the framework is deployed over edge IoT devices, ensuring low latency. Li et al. [174] have deployed a similar approach, combining both FDL and edge/fog computing to protect IIoT environments from DDoS attacks. This method also achieved high detection accuracy (i.e., 98%).

Wazid et al. [175] proposed an effective method to detect routing attacks launched by malicious neighbors, in order to target edge-based IoT environments and degrade the performance (particularly, the delay and throughput) of edge networks. This method was designed to detect routing attacks and can be deployed on edge servers to identify the suspicious nodes that launch the attacks on their neighbors. This method should be distributed on powerful servers, as the collected data would be huge, including routing messages that are sent to all the nodes in the network (i.e., broadcast messages).

Singh et al. [176] introduced a network traffic monitoring system that thoroughly inspects incoming and outgoing network packets. The proposed system specifies signature rules to detect SQL injection attacks and other traffic injection attacks, places these rules in the IDS database, compares the packets with these rules, and, if any deviation is found, the attack is detected. This method only detects one family of attacks: Traffic injection attacks. This kind of method belongs to misuse intrusion detection systems. The biggest issue with intrusion detection systems in this category is their lack of ability to detect novel attacks (i.e., attacks with no signatures in the database). The only solution is to update the signature rules placed in the database through historical attack data analysis, which takes time and effort.

Yan et al. [177] presented a multi-layer framework to mitigate DDoS attacks. The framework collects network traffic at the cloud computing layer, classifies the traffic, and detects DDoS attacks based on the captured traffic. The authors utilized a data analysis mechanism located at the cloud computing layer to inspect the DDoS attack behavior. Consequentially, the inspection information is forwarded to the fog computing layer to mutually combat DDoS attacks.

Zhou et al. [141] proposed a fog-based technique to mitigate DDoS in IIoT environments. The proposed system captures network traffic and analyzes it offline using VNFs in a local server. The analyzed network traffic information is matched with information captured at the cloud servers, to effectively detect and defend against DDoS attacks. The proposed method was designed to improve the response time and enable IIoT resource-constrained devices to efficiently adopt this technique without noticeable computational overhead. This approach consists of three levels and was implemented utilizing the Mero control system to achieve acceptable results. These methods were also designed to only detect one family of attacks (i.e., DDoS attacks), so they do not constitute a complete protection solution for IIoT environments.

Bhardwaj et al. [140] proposed a proactive technique to mitigate DDoS attacks. The proposed method uses three components to effectively detect DDoS attacks: Locally deduced information, edge function, and web service. This approach is distinctive, as the detection is accomplished in real-time and provides defense responses. The authors claimed that the proposed solution could detect IoT DDoS attacks faster than related approaches by 10 times. Additionally, the authors claimed that the proposed approach could reduce the damaging impact of DDoS by 82%.

Simpson et al. [178] proposed an approach based on fuzzy logic to detect cooperative attacks (i.e., a type of black hole attack) targeting edge nodes in IoT environments. The authors presented a trustworthy infrastructure placed on the edge, to mitigate security risks in smart cities. This infrastructure was designed to detect malicious threats (cooperative attacks, in particular) in real time. The authors position the detection mechanism on the edge computing platform to reduce the computational overhead on IoT devices. Compared to services provided by the cloud, placing the detection method at the network’s edge can decrease bandwidth utilization and delay. Once an attacker is detected, the node that launches the attack is isolated. The authors also proposed utilizing a reaction-based trust evaluation, which generates a reputation value to re-analyze suspicious entities. The proposed framework was evaluated, demonstrating its effectiveness in detecting cooperative attacks.

Zaminkar et al. [179] presented a defense technique based on node rating and ranking to deter sinkhole attacks from affecting IoT devices. The authors conducted real experiments in industrial premises containing IoT devices and launched real-world sinkhole attacks using relevant tools. The authors captured real data frames flowing from and to IoT devices communicating with the APs through Wi-Fi (i.e., traffic transferred through wireless communication). Other network traffic transferring from the APs to a central switch and then to a router was captured as well (i.e., traffic transferred using wired communication). Network traffic was captured by switch port mirroring and the Wireshark sniffing tool. The authors deployed nine commercial IoT tools in the industrial environment, which acted as infecting devices, and formed two botnets to launch the sinkhole attacks.

Khan et al. [180] introduced a smart communication mechanism that detects and prevents Sybil devices from targeting IIoT devices in PEC. Once the device masquerades as one of the IIoT devices (i.e., spoofs its identity), the adversary’s identity is detected, and a notification is sent to edge servers to deter upstream messages transmitted from that suspicious node. The building block of the proposed framework is the parallel ABC algorithm, which determines the optimal network configuration for IIoT devices on each edge server once the attack is detected. Then, the server carries out job migration with the servers nearby, in order to improve the network performance and for load balancing, based on the capabilities of the nearby servers (e.g., storage and processing capabilities). The authors conducted an experiment to validate their detection and prevention techniques, proving that the technique is capable of detecting Sybil attacks and the delay can be reduced, the throughput could be improved, and the data communication of IIoT devices in PEC could be controlled with the help of the parallel ABC algorithm.

Lawal et al. [181] proposed a fast and accurate anomaly- and misuse-based method to mitigate anomalies in IoT environments using fog computing. To ensure that an intruder is detected rapidly, the authors placed a list of IP addresses belonging to suspicious devices in a database (the signature-based part of the proposed system). Meanwhile, the anomaly detection part of the proposed framework adopted a machine learning technique known as extreme gradient boosting to differentiate malicious packets from genuine ones. The signature-based part was shown to be effective, in terms of detection time, when tested on a dataset (i.e., its detection time was faster than the anomaly detection part by more than six times). The anomaly-based part of the framework also demonstrated its effectiveness, achieving a 99% average accuracy and a 97% average recall.

Alharbi et al. [182] introduced a neural network architecture, called local–global best bat, to detect botnet attacks in the IIoT paradigm. The proposed method efficiently chooses feature representations and hyperparameters extracted from nine off-the-shelf IoT devices affected by attacks launched from two botnets: Mirai and Gafgyt. The bat’s velocity in the swarm is reformed using the local–global best-based inertia weight. Additionally, the authors utilized a Gaussian distribution in the population initialization step, in order to overcome the bat algorithm swarm diversity problem. The Gaussian density function in each generation is followed by a local search, thus accomplishing ideal exploration. The authors used a publicly available dataset (i.e., N-BaIoT) to validate their approach. This dataset consists of eleven classes: ten classes representing botnet attacks and a benign class. The proposed model was shown to be superior, compared to existing weight-optimization techniques such as PSO, achieving an accuracy of 90% in multi-class classification.

Nguyen et al. [183] adopted a dynamic analysis technique to enhance graph-based features and, hence, improve the IoT botnet attack detection performance. Printable string information is gathered using dynamic analysis when carrying out the instances. Consequentially, to traverse the graph, the printable string information is effectively employed, based on static analysis, to obtain graph-based features and eventually differentiate benign instances from attack instances. The proposed method was evaluated using a dataset of 8330 samples, including 5531 attack samples and 2799 normal samples. The method yielded a promising accuracy of up to 98.1%.

Alqahtani et al. [184] presented a feature selection method based on the Fisher score (A representative filter-based technique employed to select important features and ignore insignificant features through the minimization of intra-class distances and maximization of inter-class distances) and an IoT botnet attack detection technique based on XGBoost. The Fisher score-based feature selection method was utilized to choose the most important feature out of 115 available features, and the XGBoost-based method was used to distinguish between IoT botnet attacks and normal traffic. The authors conducted various experiments on the N-BaIoT dataset and evaluated their approach, using 10-fold cross-validation and holdout methods. The proposed feature selection method reduced the feature set to three important features out of 115 available features, thus reducing the detection time, while the selected features along with the proposed detection technique improved the detection accuracy when compared to the case where the baseline features were used.

Arshad et al. [185] introduced a lightweight IDS designed for the IoT paradigm, which best fits the requirements of constrained IoT devices. The proposed method can be implemented on IoT devices and edge routers collaboratively to improve detection accuracy, decrease false positive rates, and enhance visibility. The authors created attack signatures and placed them in a database; this database is then installed on IoT devices. Thus, each IoT device is equipped with a signature-based IDS. Furthermore, the edge-router learns the normal activities of the IoT devices, in order to detect any activity that deviates from the normal traffic. Thus, an anomaly-based IDS is positioned at the edge router. The effectiveness of the proposed solution was demonstrated, in terms of energy and memory consumption.

Arshad et al. [186] designed a similar framework for energy-constrained IoT devices, which can detect intrusions in IoT environments. The proposed framework can be implemented on IoT devices utilizing the Contiki operating system and on edge devices, in order to protect IoT environments against increasing threats (particularly, botnet attacks), while considering their low energy consumption, less computational overhead, and minimum communication cost. As with the previous approach, the proposed method installs a signature-based IDS in the IoT devices while placing the anomaly detection IDS at the edge router. Each IoT device has three mechanisms: Network monitoring, system monitoring, and detection engine. The anomaly detector consists of two GDEs and three capabilities: Detection, correlation, and alert capability. The framework’s efficacy was demonstrated, in terms of minimizing energy consumption and memory utilization.

However, the two previous approaches suffer from the following shortcomings: signature-based IDS could pose an issue for resource-constrained devices, due to the increasing number of attacks that need to be placed in the database and managed by those constrained devices. Additionally, new attacks should be added to the database; however, updating the database on each IoT device is cumbersome and consumes energy and memory resources. Moreover, the edge router is traditionally designed to forward the network layer datagrams (i.e., it processes the network layer header); however, to deploy an IDS on the edge router, it is necessary to decapsulate the packet to see the payload information, which violates end-to-end communication (i.e., the data should be transferred from the transport layer of the sender to the transport layer at the receiver).

Zhang et al. [187] presented a method to prevent signature forgery attacks in IIoT environments using a robust certificateless signature mechanism. The security of the proposed method was verified, and its effectiveness against malicious third parties and public key replacement threats was demonstrated.

Qi et al. [188] proposed a prevention scheme utilizing secure access control to ensure the security of data transmission (i.e., to prevent malicious data transmission issues) in the IIoT paradigm. The introduced technique relies on a ciphertext policy attribute-based encryption mechanism, which enables IIoT entities to apply fine-grained policies to coordinate access to IIoT data. The computational overhead of implementing the proposed technique on IIoT devices is reduced through the use of a hybrid cloud infrastructure, which handles the encryption and decryption processes. This method can also provide a new privacy capability to IoT data, known as item-level data protection; a capability that can deter key leakage issues.

Tajalli et al. [189] adopted an average consensus-based mechanism to provide smart microgrids (i.e., an IIoT application area) with optimal scheduling for real-time operations and to resist DoS attacks. The proposed method utilizes a fog layer to decrease delays and supply the necessary data storage and internal computation capabilities for the IIoT environment. The security of the proposed method was also tested in heterogeneous IIoT devices against various attacks (DoS attacks, in particular), in order to evaluate the method’s performance in the context of such attacks. Their simulation results indicated the framework’s effectiveness, in terms of accuracy, rapid response time, and feasibility.

### 7.2. Perception Layer Security

Edge nodes are resource-constrained: they are equipped with memories with limited storage capacity and micro/processors with limited data processing capabilities. Usually, these devices temporally sustain data transmitted by IIoT devices. Therefore, the complexity of data management is decreased; however, data security challenges (e.g., data leakage) may occur. Secure data storage is one of the hot topics relating to IIoT device deployment in the edge computing research area. As shown in Table 5, some solutions have been proposed recently to overcome such challenges.

Liu et al. [190] introduced a framework to preserve data storage security utilizing a privacy algorithm known as local differential and a combined AES-RSA encryption technique. The authors adopted the encryption technique to jointly and efficiently protect the secrecy of the data while making it possible to recover the data in a secure manner (i.e., an entity with the appropriate key can recover the data). This framework consists of three layers: Local, cloud, and fog. However, the proposed approach utilizes the RSA encryption technique, which belongs to public key cryptography and is known to be slow.

Hi et al. [191] utilized SDN technology to capture the data storage status information and, hence, facilitate secure data storage on fog computing nodes. In more detail, this approach designs trusted domains, security policies, and collaborative working schemes in a hierarchical fashion. The ultimate aim of this large-scale secure storage mechanism is to coordinate and authorize storage requests and provide data storage status information in a distributed manner, enabling IIoT devices to store and share data securely on the edge.

Ming et al. [192] presented an efficient technique providing data privacy protection and secure data sharing, which can be deployed to protect devices that use fog computing services and resources. The proposed approach adopts an enhanced inadvertent transfer algorithm and utilizes edge low-latency services to enable vehicles to query the optimal driving route while providing these vehicles with location privacy protection and anonymity.

Xue et al. [193] introduced a secure data-sharing approach for VCC utilizing both cloud and fog computing paradigms. The proposed method was based on encryption outsourcing and fine-grained access control. The proposed framework provides the vehicles with privacy preservation and confidentiality in an efficient way; the computation overhead is securely separated from resource-constrained devices to cloud and fog servers. Additionally, response delay can be reduced while preserving the consumption of fog server resources with the help of vehicle mobility prediction and pre-pushing data to certain fog servers. The proposed method yielded a promising reduced response latency and overhead saving in edge devices.

Fan et al. [194] introduced a data-sharing technique designed for vehicular fog computing, in order to securely recover stored data. The proposed method utilizes a novel encryption method with a multi-authority ciphertext mechanism, ensuring data access control in vehicular networks. The proposed framework also integrates an effective mechanism for attribute revocation. Therefore, vehicular network systems can effectively perform attribute revocation and execute data access authorization using the proposed framework, guaranteeing data sharing with low latency.

Adil et al. [195] introduced an approach to identify jamming attacks utilizing edge nodes. The authors deployed three edge nodes equipped with different transmission frequencies in a WSN and used the RTT measurement of the transmitted signal to detect jamming attacks targeting the transmission channel. Even if one transmission channel (i.e., the one that an edge node is communicating through) is jammed, the other two edge nodes would be able to verify the wireless transmission serviceability in the WSN. Moreover, the RTT of the transmitted signal from the neighboring channel is also intermittent, compared to its usual time interval, due to interference in the neighboring channels. This interference indicates the existence of a jamming attack in the WSN. The proposed method was implemented using OMNeT++ and accomplished a detection rate of 94%.

Bany et al. [196] proposed a protocol that deals with proactive jamming attacks targeting IoT networks. This protocol relies on the channel and routing assignment, and does not require new hardware or entities installed in the network or servers. The aim of this protocol is to enhance the overall packet delivery ratio of the IoT network in the context of normal activities performed by IoT devices, multi-channel fading, and jamming attacks. The introduced method comprises three steps: Path discovery, channel assignment, and route selection. The proposed method enhanced the packet delivery ratio in IoT networks, compared to existing protocols.

Abhishek et al. [197] proposed a technique to detect jamming attacks in IoV networks. The authors mentioned that vehicular networks are vulnerable to jamming attacks, due to the nature of the shared wireless media through which the packets are transmitted. The authors focused on a type of Jamming attack in which the attacker waits until packets are transmitted, and then the attacker jams the channel. This type of attack is severe, as the packet drop rate increases and the delay of the network is noticeable. Thus, sensitive applications that demand real-time communication would be disrupted. To solve this issue, the authors introduced a detection technique based on SVM to identify jamming attacks. To train the proposed method, the authors created a dataset of packet drop probabilities obtained from jointly sufficient statistics. The proposed method was tested, and its effectiveness, in terms of detection ratio, was proven.

### 7.3. Application Layer Security

This subsection discusses the work proposed to secure the IIoT application layer. Table 6 compares those works focused on improving application layer security.

Dovom et al. [198] introduced a framework that detects and categorizes malware, especially in IoT and IIoT environments, by diverting the program’s opcodes into a vector space and adopting both fuzzy and fast fuzzy pattern tree mechanisms. The fast fuzzy pattern tree-based technique achieved acceptable accuracy and good detection time. The framework also utilizes both robust feature extraction capability and a fuzzy categorization component. These components enable the framework to become a typical edge computing method that detects and categorizes malware. The only issue with this system is its reliance on fuzzy logic, which is known to be inaccurate when predicting unseen samples.

Guizani and Ghafoor [199] presented a software-based framework that adopts NFV technology to resist malware diffusion in heterogeneous IoT environments. To deploy a precise countermeasure, the authors deployed a deep learning-based IDS to detect a broad range of malware promptly. The designed IDS is based on a combination of two well-known deep learning algorithms (i.e., RNN and LSTM). Once the malware is detected, the framework provides software or operating system updates to address the security vulnerability that enables the attacker to break into the system.

Khoda et al. [200] observed that several IDS datasets lack a balance between the classes in the training set (i.e., the number of samples for the benign class is much higher than the number of samples for the attack class), which may affect the performance of machine learning-based IDSs. Thus, the authors presented an over-sampling (a mechanism that increases the number of samples of classes with fewer samples; for example, by duplicating the samples of that minority class) technique to deal with this problem. The framework also introduces two capabilities to detect edge computing malware in a unique way. The first capability utilizes fuzzy set theory, while the second one uses a new loss function capable of dynamically prioritizing malware samples. The proposed framework accomplished superb results, compared to related techniques. The method achieved an improvement in terms of the F1 performance metric, which reached over 9% when compared to related work.

Alaeiyan et al. [203] introduced an edge layer deployable multi-label malware detection system-based fuzzy clustering. This system enables CPS networks to accurately predict malware threats. The Opcode frequencies are represented as a feature space, which is used with the proposed framework to conduct statistical analysis and differentiate malware categories. The proposed method was evaluated using three datasets, in which a high performance was achieved, in terms of accuracy.

Shen et al. [204] investigated IoT malware spread behavior to determine the best possible malware detection techniques for protecting the privacy of IoT smart objects and preventing the spread of malware. The authors introduced a joint cloud-fog infrastructure and deployed an IDS to detect malware capable of overcoming the heterogeneity of smart sub-nets and the limited resources of IoT devices. Due to the smart object malware uncertainty, the authors also applied a signaling game to reveal the communication between the IoT devices and the corresponding edge nodes. The authors also detailed some related mechanisms, such as theoretically calculating the optimal Bayesian equilibrium of the game to enhance malware identification probability. Additionally, the researchers explored the factors influencing the optimal probability of an IoT device spreading malware, as well as factors that affect the performance of fog nodes in identifying an infected IoT device. Moreover, the researchers provided a method demonstrating the practical and potential application of preventing the spread of malware in IoT networks.

Alhawi et al. [205] proposed a decision tree-based approach to detect Windows ransomware network traffic attacks. The proposed framework uses a specialized version of the decision tree, known as J48, and the authors evaluated the method using conversation-based network traffic samples (i.e., packets) along with extracted features (i.e., fields). The proposed framework achieved an acceptable true positive rate of about 97%.

Azmoodeh et al. [206] proposed an approach to detect ransomware attacks targeting IoT networks by measuring the power consumption of Android devices. The proposed method measures various processes to scan energy consumption patterns and differentiate ransomware attacks from legitimate applications. The authors compared four well-known machine learning algorithms (i.e., SVM, neural network, *k*NN, and random forest) using a dataset collected from VirusTotal API (This dataset can be found at the following website: https://www.virustotal.com/gui/home/upload (accessed on 20 March 2023)). The authors conducted various experiments to compare the machine learning algorithms and fine-tune the number of neighbors hyperparameter, in order to achieve the best result possible. *k*NN with DTW capability achieved the best results, in terms of accuracy, recall, precision, and F1 score, compared to the other machine learning algorithms.

Almashhadani et al. [207] presented a detailed behavioral analysis of activities occurring when crypto-ransomware—in particular, a type of severe ransomware known as Locky—attacks a network. The authors built their own test bed to validate their assumption. They extracted some important features from the network packets, to classify the captured traffic into various types. Additionally, the authors presented a network-based IDS, utilizing two separate detectors working simultaneously at two levels: Flow and packet. Various experiments were conducted using the features extracted by the authors and four machine learning algorithms: Random forest, decision tree, naïve Bayes, and SVM. The proposed technique was shown to be effective in detecting ransomware attacks, through five performance metrics (accuracy, false positive rate, precision, recall, and F1 score), and provided an outstanding detection rate and low false positive rate. The best machine learning algorithm in the packet-based set of experiments was the decision tree, yielding 97.92% accuracy, 97.9% precision, 97.9% recall, 97.9% F1 score, and a false positive rate of 0.021. Meanwhile, the best machine learning algorithm in the flow-based set of experiments was naïve Bayes, which obtained 97.08% accuracy, 0.029 false positive rates, 97.72% precision, 97.71% recall, and 97.71% F1 score.

Maiorca et al. [208] introduced an Android ransomware attack detector using the random forest ensemble method. The proposed technique differs from previous methods, in that it utilizes extracted features from API packages to categorize applications, without needing to be familiar with user-defined content (e.g., strings) and the language used to write the application. The authors evaluated the proposed approach on two public datasets (i.e., the ransomware dataset (As indicated by the authors, this dataset can be found at http://ransom.mobi/ (accessed on 25 March 2023)) and the malware-trusted dataset (Found at https://www.sec.cs.tu-bs.de/~danarp/drebin/ (accessed on 25 March 2023))). The results indicated that the proposed approach is applicable, with very high accuracy, to differentiate malware from Android ransomware attacks. Additionally, the authors flagged the detected ransomware applications utilized by the VirusTotal service.

Sgandurra et al. [209] introduced a dynamic analysis and classification approach based on logistic regression, which identifies ransomware threats when users install applications. The introduced method scans some actions executed by applications at the time of installation, in order to detect any indication of ransomware activity. The authors validated the technique on a dataset consisting of 583 ransomware samples (downloaded from the VirusShare website) belonging to 11 classes and 942 samples belonging to normal applications. The authors compared their technique with naïve Bayes and SVM. The proposed method was found to be superior to the other methods, in terms of the low complexity of the underlying machine learning algorithm and detection rate (achieving 96.3% detection rate and 99.5% ROC curve).

Tseng et al. [210] proposed a DNN-based approach to identify ransomware in a timely manner. The authors presented a labeling mechanism and chose some significant features in order to improve the performance of the proposed method and reduce the detection time. The proposed method achieved an acceptable detection rate and false negative rate.

Ogundokun et al. [211] proposed a detection technique based on machine learning to identify ransomware attacks targeting IoT devices. Experiments were conducted using a laptop computer, a projector, and an Android device. Along with detecting ransomware attacks, the proposed system monitors the power consumption of IoT devices operating processes every 500 ms, using Power-to-track. The proposed method achieved acceptable performance in four metrics: Accuracy, recall, precision, and F-score.

Al-Hawawreh et al. [212] conducted a comprehensive systematic analysis of ransomware attacks targeting IIoT devices, and suggested several potential defense mechanisms. The authors deployed IIoT devices in an industrial setting following IIRA and analyzed the shortcomings of IIoT environments that might be exploited by ransomware threats. The test bed contained I/O devices (i.e., actuators, sensors, and controllers), virtual components (i.e., mail servers, cloud servers, maintenance operators, and SCADA monitoring devices), and IIoT gateways. The authors found that the gateways in the IIoT networks are susceptible to ransomware threats, where IIoT devices and systems might be affected through gateways. The IIoT gateways share some default capabilities; they can act as mediators between the outside world and the IIoT environment (i.e., I/O devices or PLCs). Full access to the IIoT gateway can be gained once an attacker initiates a ransomware attack targeting that gateway, changes the legitimate gateway’s credentials, and updates the firmware with malignant software. Therefore, the malicious gateway would reveal any data transmitted from users to the external world (or vice-versa). Consequentially, the authors launched ransomware attacks in the considered IIoT environment, utilizing Python scripts similar to the Erebus Linux Ransomware attack. Furthermore, the authors suggested some potential detection and defense mechanisms to protect IIoT environments against ransomware attacks, including the adoption of next-generation firewalls that contain enhanced traffic filtering mechanisms, the utilization of monitoring systems (e.g., IDSs) to detect attacks as early as possible, and the placement of IIoT edge gateways in a trusted zone to prevent infected gateways from affecting the IIoT infrastructure.

To summarize this section, we can make some observations related to the state-of-the-art methods. Devices, networks, and exchanged data between devices could all be targeted by cyber-criminals in various communication systems. However, the difference when securing the deployment of IIoT devices in edge or fog computing is that the significance of edge security expands when the data are downgraded to edge devices. The traditional protection of the exchanged data between IIoT edge devices, edge computing-based IIoT networks, and the devices themselves is low, while the complexity of the network that involves both heterogeneous IIoT devices and edge servers is high. Thus, proposing and standardizing new approaches that protect edge networks or data sharing is difficult, particularly when considering methods that require changes in the hardware, standardized communications protocols, or existing infrastructures.

For those approaches that do not impose changes to the hardware, communication protocols, or existing edge network infrastructure—for example, IDS approaches that detect various edge computing IIoT attacks such as injection attacks, DDoS attacks, and routing attacks—it is necessary to provide a solution that is lightweight and accurate. In this line, the proposed solutions for secure data sharing need to be further improved and investigated. These solutions are still limited and may become a hot topic in the near future. The use of emerging technologies, such as Blockchain and AI, could add value to the secure data sharing and management research area.

Most of the IIoT network layer security solutions are detection-based. Most IIoT network layer security solutions utilize machine learning to detect attacks such as DoS that prevent the IIoT devices from accessing edge nodes (i.e., violate the availability requirement). The detection accuracy and time of these approaches are decent; the accuracies of these approaches can range from 90% to 100%, depending on the dataset and data division, and can detect intrusions in real time. A few proposed solutions mitigate security issues that violate confidentiality and data integrity. These solutions rely on well-known encryption mechanisms to mitigate the impact of some security issues, such as malicious data transmission transferred from IIoT devices to the edge nodes or vice-versa and signature forgery attacks.

The majority of the IIoT perception layer security proposed solutions prevent/mitigate security challenges that violate confidentiality, secure data sharing/storage security, and privacy. Some of these approaches rely on standardized encryption methods such as AES and RSA to provide confidentiality to the transmitted data from IIoT devices and edge nodes and vice-versa and to preserve the security of the data stored at the edge node. Infrequent solutions utilize machine learning to detect jamming attacks that violate the availability requirement, targeting the communication links between the IIoT devices and edge nodes.

Most IIoT application layer security solutions are detection-based. These solutions utilize machine learning to detect attacks that inherited traditional networks and IoT environments, such as malware and a subtype of malware known as ransomware. These attacks violate integrity, confidentiality, and authentication. Thus, detecting these attacks might help security personnel take further countermeasures to prevent these attacks from spreading to the IIoT devices (especially if they control the edge nodes). These approaches’ accuracies are reasonable, ranging from 70% to 99.5% depending on the used dataset.

## 8. Opportunities and Future Directions

Individuals and organizations have begun to appreciate the proficiency fog and edge computing paradigms provided to the internet community. This appreciation extends the utilization of these paradigms to store, communicate, and process resources through edge/fog networks instead of CC. The advances of IIoT secure deployment on edge computing were investigated in the previous section. After exploring the progress in the state-of-the-art, there were still some deficiencies in several proposed techniques, hindering the solution of the corresponding security issues. Emerging technologies, such as AI and edge computing, provide various opportunities and issues when integrated into secure IIoT environments. However, the scalability and resilience of edge and fog computing involve various security and privacy challenges [1,213], which may be further investigated by researchers. This section discusses some opportunities and challenges for the secure deployment of IIoT devices at the edge, including secure data sharing, security monitoring, and authentication and access control. This section also presents some insights into how the security of IIoT might be advanced with the help of edge/fog computing and AI in the near future.

### 8.1. Secure Data Sharing

IIoT devices generate a huge volume of real-time data; thus, data mining enables industries to make the right decisions and inevitably enhance their production efficiency [214]. Traditionally, the design of IIoT is mostly vertically supplied by closed applications, which enable industries to enhance manufacturing processes in a single site. Thus, data islands are forms that need to be split, through the utilization of edge computing, in order to improve their flexibility. Secure data sharing is a complicated issue. Subdividing data islands in an efficient manner and sharing real-time data generated by IIoT devices among heterogeneous applications and entities securely and in a timely manner is expected to become a hot topic related to the IIoT-based edge computing paradigm. Sharing data via edge computing faces two key issues: The limited performance of edge devices, which makes it hard for robust security techniques to be applied, and the unavoidable huge amounts of data, which may lead to more serious consequences (e.g., destruction and cyberattacks). In this context, data generated by IIoT devices in edge computing can be securely shared through the use of a blockchain [215]. Additionally, the unavoidable huge amounts of data could be preprocessed by machine learning techniques to extract the meaning features and feed these features to the edge devices for processing purposes.

If an IIoT device wants to store sensitive data securely, resource-constrained devices such as IIoT devices may not have the storage capacity and processing capability to enable them to store data securely. Thus, edge computing provides IIoT devices with a solution. The edge computing nodes can be equipped with sufficient computational (to deal with complex encryption methods) and storage capabilities, which enable IIoT devices to store data and share data securely. Moreover, edge computing nodes can be distributed close to the IIoT devices, decreasing the associated latency. Even when big data are generated from IIoT devices (i.e., those which cannot be processed or stored at the edge node), the edge computing node could act as a server to the IIoT devices and a client for cloud computing servers, facilitating data storage and processing (the edge node can also transparently encrypt or decrypt data stored in the cloud server) [216,217]. Furthermore, since the edge nodes are close to the IIoT devices (i.e., in the same local area network), data transmitted between the IIoT devices and edge nodes will never leave the network premises, so complex encryption mechanisms are not necessary. Therefore, IIoT devices would consider the service to be provided by the edge node and do not need to be aware of the corresponding security or storage methods.

As edge nodes can act as gateways between IIoT devices and external devices, industries can secure and control data flow to and from external devices. By setting up an edge node, high-security standards can be maintained, mutual authentication with outside work can be accomplished, and the limited capacity of IIoT devices can be overcome; hence, IIoT devices only need to process secure communications with edge nodes. Additionally, the edge node can activate the data flow strategy, in order to gain access to the content of the message when processing the traffic passing through it [218].

The distributed service nature of edge and fog computing might lead to data leakage; therefore, it is necessary to prevent unauthorized parties from disclosing stored or in-transit data. Therefore, light encryption techniques such as cryptographic hashing and homomorphic encryption methods could be utilized to protect the transmitted data stored at distributed locations from disclosure. Encrypted data prevents disclosure even if the attacker intercepts the data in transit or accesses secured data stored in specific servers.

Data exchanged between IIoT devices, or between IIoT devices and edge nodes, should be transmitted securely, in order to prevent intruders from modifying or altering the data even if interception occurs. Cryptographic signature verification systems are notable techniques used to enforce integrity on exchanged data, similar to the GnuPG technique (found at https://gnupg.org/ (accessed on 2 April 2023)), which is utilized to sign transferred data digitally at the sender side and verify it at the receiver side. Data integrity is of paramount significance to IIoT devices when utilizing services on edge computing as, in this situation, the communication between the network entities depends entirely on the network, considering the distributed nature of the network topology.

### 8.2. Security Monitoring

Edge computing platforms can be equipped with various capabilities, in order to satisfy the needs of IIoT environments. Thus, they may serve as the perfect candidate to be equipped with a substantial system capable of monitoring potential security threats.

An edge node can be equipped with an IDS capable of storing signatures of well-known attacks, thus having the ability to detect intrusions from captured traffic based on these signatures. In the case that the IDS is based on machine learning/deep learning and a number of attack samples need to be used to train the machine learning algorithm, a cloud server could be utilized to train the algorithm, and the weights could be transferred to the edge node for the detection intrusions; hence, the latency issues associated with cloud solutions can be addressed [219].

As sensors communicate their measurements directly to edge nodes, edge nodes can execute anomaly detection mechanisms to ensure that the measurements are within an acceptable range. Therefore, a complete security monitoring system could be deployed to monitor data passing through the edge computing platforms to and from the IIoT environment, allowing for the detection and deterring of threats [220,221,222]. Additionally, actions can be taken based on traffic passing through, in order to mitigate DoS or DDoS attacks targeting IIoT environments or the edge computing infrastructure.

DoS attacks are among the primary issues restraining the availability of services from authorized IIoT devices. These issues could be partially resolved by edge/fog computing, due to the distributed nature of the computational resources. However, DDoS could degrade or prevent authorized IIoT devices from accessing these services. IIoT environments could deploy smart DNS resolution services, WAF, and other smart network traffic monitoring and filtering techniques to ensure that services are always available.

### 8.3. Authentication and Access Control

Edge computing can address diverse authentication issues. Edge computing can be applied to replace solutions that necessitate the need for third-party servers [223,224,225,226,227]. An edge node is not resource-constrained and can perform complex computational tasks, consequently having the ability to act as a third-party service to provide IIoT devices with the required authentication mechanism. One advantage of edge nodes over third-party servers is their on-premise placement, providing IIoT devices with low latency when the devices and edge nodes exchange authentication messages. Moreover, an edge node can act as a certificate authority (An organization responsible for signing, storing, and issuing digital certificates) for IIoT devices, thus improving upon conventional PKI infrastructures. Therefore, edge nodes can form a peer-to-peer network to establish a united and powerful key infrastructure [10].

Edge nodes could also serve as trusted gateways, adding new IIoT devices, removing existing ones, and being responsible for re-keying. Some existing works have detailed devices with the required authentication ability through various types of servers, including NFC tags [228,229,230], smart cards [231,232,233], RFID tags [234,235,236,237,238], and biometric traits [239,240,241]. Edge nodes could become a substitute for such a server, and may be attached to sensors, acting as proxies for sensor measurements. In this context, the scalability could be expanded, as more fog nodes may be distributed such that they are reachable by close IIoT devices. Therefore, if there is an urgent need to apply authentication for device updates utilizing NFC keys or biometrics, maintenance engineers would need only the fog node and its binding to the associated key, rather than searching for each device related to the keys independently.

Other authentication capabilities could be brought to IIoT environments through edge computing, such as TPM and TEE. This can be accomplished by setting up a secure communication tunnel between the TEE or TPM and the edge node, as well as adopting a key setup protocol equipped with a one-time pairing feature. Thus, future work is expected to integrate trusted capabilities with edge computing platforms.

Similar to the opportunities related to authentication, access control could be enhanced when integrated with edge computing to authorize IIoT devices. Resource-constrained devices are not ideal places to carry out access control policies on; thus, these policies could instead be relocated to edge nodes. This would introduce a new issue (i.e., centralization, which might lead to SPOF), as all access control policies would be outsourced from IIoT devices to a specific edge node. A possible solution to this issue is to distribute access control policies through multiple edge nodes, in order to prevent the possibility of SPOF.

IIoT devices share the secure and reliable services provided by edge and fog computing, so mutual trust between IIoT devices and between IIoT devices and edge/fog nodes should be considered. This compels edge and fog servers to prove that the edge nodes that exchange messages with IIoT devices are trustworthy and that their services are genuine. Meanwhile, edge nodes must ensure the authenticity of the IIoT requesting services from edge nodes. These challenges may lead the research community to develop mutual authentication models for IIoT devices and edge nodes utilizing lightweight techniques/devices such as Trust Chain [242] (an authentication scheme based on a permission-less Blockchain network) and PUF [243] (an object that provides a physical component with a trust anchor or an unrivaled fingerprint by exploiting the intrinsic randomness introduced during production), or developing techniques with low complexity to be deployed in IIoT environments.

### 8.4. Maintainability and Resilience

Industrial systems can benefit from edge computing in terms of maintainability. Since most essential industrial systems are connected to the internet, this connectivity enables brisk software management and updates. However, it is preferable to utilize edge computing devices to ascertain the validity of these updates and run thorough tests (e.g., running the updates needed for the industrial systems at the edge in a sandbox environment to ensure no anomalies reside before forwarding them to the industrial devices) [10]. This practice also assures the continuity of services at the industrial systems, enabling them to perform their routine tasks without degradation. Additionally, edge computing devices could act as hubs for industrial systems where industrial devices can view software information, including the version number and required updates, or manage configuration files for industrial devices. Since edge computing devices can provide an easily accessible place to read NFC tags or other authentication modules, improving industrial systems maintenance procedures and other authentication factors is feasible without attending to each device for maintenance independently and physically.

Edge nodes can act as security agents capable of disabling and isolating compromised industrial software. This practice enables network administrators or security personnel to scrutinize the affected software thoroughly while the industrial devices perform other routine tasks. Additionally, edge computing can overcome intermittent internet connectivity to cloud services. Edge devices can be provisioned with convenient processes usually performed in the cloud and requested frequently by industrial systems. Therefore, the edge node could provide the services if the connection to the cloud server fails. Also, industrial devices sometimes send data to the cloud for processing purposes, so that intermittent connectivity would be an issue. Thus, edge computing could be a medium to store or forward data from its end to the cloud. In this manner, the edge node can buffer information even if there is no internet connectivity and transmit the buffered information when the internet connectivity is restored. Thus, industrial devices would be minimally affected by intermittent connectivity.

## 9. Conclusions

As a modern industrial solution, IIoT connects network components using advanced communication technologies, helping industries to monitor, exchange, collect, and analyze data, thus simplifying crucial decision-making problems, improving productivity, and significantly enhancing performance more than ever. Edge computing can be adopted in the IIoT to process a portion of the large-scale real-time sensing data on the network’s edge, near the origin of the data. In this way, the limited transmission bandwidth and long-delay decision-making (i.e., if cloud computing is employed) issues may be resolved. In this survey, a review of IIoT attacks, requirements, and solutions that utilize AI and edge computing, with a focus on the period from 2017 to 2023, was conducted. The security challenges were classified into three categories, based on the IIoT security layer: application layer threats, network layer threats, and perception layer threats. We identified twenty-two attacks that may target these IIoT layers: four attacks targeting the perception layer, eight attacks targeting the network layer, and ten attacks exploiting application vulnerabilities. Each attack was linked with the security requirement it violates and common countermeasures that could be taken to prevent the attack. Additionally, solutions proposed to detect or prevent these attacks and to generally improve the security of IIoT were discussed. Moreover, challenges encountered in the IIoT field when adopting edge computing and AI were detailed, along with the opportunities these technologies provide. Finally, future research directions were proposed, providing researchers with insights into utilizing AI and edge computing to secure the IIoT paradigm. Although edge computing presents various advantages to the IIoT environment, it raises new overhead for maintenance personnel. It might mandate special training to more network administrators that belong to the organization, making edge computing more expensive than cloud computing, which can be maintained by experts on the service provider’s side. Additionally, in case of a security breach found in a specific edge software, the maintenance team would be responsible for patching every software installed on distributed edge devices compared to updating software only on cloud computing infrastructure by security experts at the service provider’s end.

## Figures and Tables

**Table 1:** Comparison of the main characteristics of IoT and IIoT.

Characteristic	IoT	IIoT
Application examples	Smart home, health monitoring, indoor localization	Smart transportation, intelligent logistics, smart manufacturing, remote maintenance
System Framework	Self-reliant	Industrial facility-reliant
Delay sensitivity	High	Low
Mobility	High	Low
Deployment size	Small	Large
Deployment preciseness	Low	High
Data volume	Medium	High

**Table 2:** Comparison of the related literature.

Scope	Ref.	Major Contribution	Advantages	Limitations
IoT security	[32]	A comprehensive overview of IIoT security threats	Attacks perfectly linked to security requirements	The role of emerging technologies in securing IoT networks is not discussed
	[33]	A detailed review of IoT threats and vulnerabilities	A distinctive categorization of IoT vulnerabilities and a discussion of about 100 research ideas	Attacks are not completely linked to security requirements and the the impacts of integrating emerging technologies on IoT security are not discussed
	[34]	An overview of security requirements for several IoT application domains	Noteworthy security requirement prioritization for each application domain	The depth of the challenges section is minimal
IIoT security	[35]	A comprehensive review of cyberattack classes	Outstanding future directions and potential applications are discussed	Cyberattacks are not linked to security requirements and the impact of emerging technology on IIoT security is not discussed
	[6]	A survey of challenges faced by Industry 4.0 environments	A unique overview of challenges related to energy adequacy, interoperability, and security	Security requirements and emerging technologies impact are not discussed
	[36]	An overview of IIoT security solutions	A unique description of the building and linking of IIoT devices with security in mind	The depth of the survey is minimal
	[37]	A unified architecture format of security requirements in IIoT	A detailed comparison of security requirements within heterogeneous	The authors discuss a few use cases of IIoT devices
	[38,39]	A discussion of IIoT security requirements	A comprehensive overview of solutions that deal with security violations	The depth of the review is minimal
	[40]	An overview of IIoT security, threats, and countermeasures taken by industries	A distinctive categorization of the IIoT, exploration of countermeasures taken by industries utilizing security requirements	Lacks discussion of the role of adopting emerging technologies to protect the IIoT paradigm
	[41]	An evaluation of emerging IIoT security challenges and investigation of existing countermeasures	An ideal mapping study between challenges and countermeasures is presented	The survey is not comprehensive; it complements existing related work
	[42]	A review of IIoT security threats and AI and Blockchain-based solutions	A distinctive synopsis outlining the advantages and disadvantages of each solution is introduced	Lacks detailed security threats discussion and enough blockchain and AI background, solutions comparison elements are brief
Edge security	[43]	An overview of the IoT Edge paradigm and applications	An investigation of opportunities provided by edge computing to improve IIoT security	The depth of the overview is minimal
	[44]	A thorough discussion of fog computing security and privacy issues	A distinctive observation related to the unsuitability of methods used to secure CC for fog computing is introduced	The depth of the discussion is minimal
	[45]	A detailed tutorial of the edge computing paradigm	Incandescent solutions to privacy and security are thoroughly discussed	The connection between edge applications, threats targeting them, and security solutions is missing
Edge Computing in IIoT	[46]	A roadmap for smart manufacturing to integrate IoT and edge computing	One of the first surveys to discuss this area	Security requirements and challenges are inadequately discussed
	[47]	A demonstration of two scenarios of how IIoT benefits from fog computing	A unique comparison of cloud and fog computing when integrated with IIoT	The overview is scenario-specific (i.e., not comprehensive)
	[48]	An overview of edge computing reference architectures in IIoT	A comparison of reference architectures is presented	The depth of this overview is minimal
	[49]	A discussion of the integration of fog computing and IIoT	Two enabling technologies that can add value to the integration are uniquely discussed	This survey is not comprehensive
	[50]	A review of edge computing and IIoT integration	A discussion of recently proposed solutions, recent challenges and few use cases	The lack of in-depth discussion of security challenges and sufficient application examples
	[8]	Discussion of the Industrial Revolution background and transformation enabling technologies	A well-organized and thorough discussion of communication and network protocols	The discussion part of edge computing lacks essential details
	[21]	A review of current solutions related to adopting edge computing into IIoT	Distinctive technical details of some significant edge services that add value to the IIoT paradigm	Security opportunities brought when integrating edge computing into IIoT is partially discussed
Secure IIoT-Edge	[10]	A systematic survey of IIoT security from 2011 to 2019	A thorough discussion of IIoT security challenges, requirements, and opportunities provided when adopting edge computing that could secure IIoT paradigm	The IIoT attacks are not deeply discussed and th depth of the opportunitiese part is not sufficient
	**Ours**	A thorough categorization of IIoT attacks, security requirements, and security benefits from integrating edge computing and IIoT	A distinctive linkage of IIoT attacks and requirements is introduced and research attempts to overcome security challenges (with a focus on the period 2019–2022) are comprehensively discussed	N/A

**Table 3:** Popular security threats that target the three IIoT layers and their common countermeasures.

Layer	Attack	Violated Requirements	Common Countermeasures
Perception	Node Capture	Confidentiality, Authentication	Abolishing information related to secure keys after disassociation [102]
	Jamming	Availability	Increasing interference resistance using techniques such as FHSS [103] and DSSS [104]
	Sleep Deprivation	Availability	Ensuring security policies are not violated using policy-based IDS [105]
	Replay	Integrity	Utilizing timestamps and nonces [106]
Network	Selective-Forwarding ${}^{a}$	Availability	Detection and prevention using a combination of IDS and IPS [107]
	Eavesdropping	Confidentiality, Privacy	Employing access control and data encryption techniques [108]
	Sybil and ID Cloning ${}^{b}$	Authentication	Applying packet filtering, IDS, and localization techniques [109]
	Wormhole	Confidentiality, Availability	Deploying secure neighboring discovery techniques and measuring challenge-response and RTT delay [110]
	Denial of Service	Availability	Utilizing traffic filtering, IDS, and tracking techniques [111]
	Man in the Middle	Confidentiality, Authentication	Employing light encryption techniques and deploying IDS [112]
	Sinkhole	Availability	Employing IDS and IPS to detect and prevent this threat [113,114]
	Black hole ${}^{c}$	Availability	Utilizing various routing paths and deploying IDS and IPS techniques [115]
Application	Malicious Code Injection	Confidentiality, Authentication	Employing private-key cryptography, light public-key encryption, and authentication mechanisms [116]
	Cross-site or Malicious Scripts	Confidentiality, Authentication	Deploying signature-based IDS and content and pattern analysis techniques [117]
	Malware Injection	Integrity	Deploying IDS, IPS, and malware removal mechanisms [118]
	Data Distortion	Integrity and secure data sharing	Utilizing access control, encryption, and recovery [119] such as backup mechanisms
	SQL Injection	Confidentiality, integrity	Utilizing parameterized statements, IDS, access control, and encryption techniques [120]
	Ransomware	Confidentiality, Authentication	Employing traffic filtering, IDS, IPS, and encryption techniques [121]
	Side-channel	Confidentiality	Protection of cryptography techniques, preventing traffic analysis, and enforcing strict access control policies [122]
	Authorization and Authentication	Authentication and access control	Using access control and authentication techniques [123]

${}^{a}$ also known as gray hole; ${}^{b}$ a.k.a. malicious cloning; ${}^{c}$ sometimes referred to as selfishness.

**Table 4:** Summary of works focused on enhancing the security of the IIoT network layer.

Scope	Ref.	Algorithm	Resolved Issue	Security Requirement	Dataset	Performance Metrics
Deep learning-based IDSs	[164]	LSTM	DoS attacks	Availability	ISCX, AWID	98.22% accuracy on AWID, 99.91% on ISCX
	[165]	Stochastic MC	false injection	Confidentiality, integrity	Custom	NA
	[166]	LSTM and 1D CNN	DDoS	Availability	DoS2019	1D-CNN: 99.3% precision, 98.9% recall, 99.1% F1 score ${}^{a}$
	[167]	ANN, RNN-LSTM, RNN-GRU	botnet attacks	Availability	BotIoT	ANN: 99% accuracy, RNN ${}^{b}$: 98% accuracy
	[168]	Stacked deep autoencoders	botnet attacks	Availability	N-BaIoT	3% improvement
	[169]	LAE and B-LSTM	botnet attacks	Availability	BotIoT	93.17% (binary), 97.29% (multiclass)
	[170]	RNN	botnet attacks	Availability	BotIoT	99.75% recall, 99.62% precision and F1 score
	[171]	CFBPNN	botnet attacks	Availability	5 datasets	100% accuracy
	[172]	Custom algorithm	botnet attacks	Availability	N-BaIoT	99.76% accuracy, 99.68% F1 score, 0.2250 μ ${}^{c}$ testing time
	[173]	Federated DL	zero-day botnet	Availability	Bot-IoT, N-BaIoT	99.79% accuracy, 99.51% precision, 96.27% recall, 97.68% F1 score 99.00% accuracy, 96.87% precision, 97.24% recall, 96.88% F1 score
	[174]	Federated DL	DDoS attacks	Availability	UNSW NB-15	98% accuracy
Signature-based IDSs	[175]	Custom algorithm	routing attacks	Availability	NS2 ${}^{d}$	95.0% detection rate, 1.23% FPR ^e^
	[176]	Custom algorithm	SQL injection	Confidentiality, integrity	Custom	4.7× improvement
	[177]	Custom algorithm	DDoS attacks	Availability	Custom	Not reported
	[141]	Custom algorithm	DDoS attacks	Availability	Custom	Up to 99.84% detection rate, as low as 129 ms testing time
	[140]	Custom algorithm	DDoS attacks	Availability	Custom	Reduced the damaging impact by 82%
	[178]	Fuzzy logic	Black hole attacks	Availability	Custom	more than 90% accuracy
	[179]	Node ranking	sinkhole attacks	Availability	NS3	96.19% detection rate, 4.16% FPR, 4.04% FNR ${}^{f}$
	[180]	Parallel ABC	Sybil attacks	Authentication	Simulation	≈ 97% accuracy, 97% sensitivity
	[181]	XGBoost	botnet attacks	Availability	BoT-IoT	99.99% accuracy, 97.5% recall, 99.5% precision, 98.5% F1 score
	[182]	Gaussian distribution and local search	Mirai and Gafgyt botnets	Availability	N-BaIoT	90% in multiclass classification
	[183]	Dynamic analysis	botnet attacks	Availability	Custom	98.1% to 91.99% accuracy
	[184]	Fisher score and XGBoost	botnet attacks	Availability	N-BaIoT	99.96% average accuracy
	[185,186]	Custom algorithm	DoS attacks	Availability	Custom	A supply voltage ranging from 2.1 to 3.6 V
	[187]	Certificateless signature mechanism	signature forgery attacks	Integrity	NA	NA
	[188]	Ciphertext policy attribute-based encryption	malicious data transmission	Confidentiality, authentication	NA	NA
	[189]	Average consensus-based mechanism	DoS attacks	Availability	Matlab	Average calculation time of 4.7 ms per 100 iterations

${}^{a}$ The results of the LSTM-based approach were less accurate; thus, the better-performing method is reported in this cell; ${}^{b}$ both RNN-LSTM and RNN-GRU have identical accuracies; ${}^{c}$ this symbol represents time in microseconds format; ${}^{d}$ NS stands for network simulator; ^e^ false positive rate; ${}^{f}$ false negative rate.

**Table 5:** Summary of the works focused on enhancing data sharing and storage security.

Ref.	Method Characteristics	Provided Security Requirement	Advantages	Limitations
[190]	Hybrid AES-RSA	Confidentiality, secure data sharing	It efficiently protects the secrecy of the data and enables devices to recover the data in a secure manner	It relies on RSA (i.e., an asymmetric encryption method), which is slow
[191]	Hierarchical and distributed	Secure data at rest, while providing IIoT devices with status updates	A secure method capable of large-scale information and data storage.	It is not linked to data and infrastructure characteristics
[192]	Combining a super-increasing sequence and modified oblivious transfer	Privacy, secure data sharing	It efficiently provides secure data sharing and anonymity	It is centralized
[193]	Encryption outsourcing and fine-grained access control	Secure data sharing, access control	It achieves encouraging response latency reduction and overhead savings for edge devices	The security analysis was not discussed in detail
[194]	Encryption with multi-authority cipher-text	Secure data at rest	Data access authorization and secure data sharing are ensured to protect edge devices against collusion attacks with low delay	The high scalability of edge networks might cause other security issues to emerge
[195]	Based on an anonymous edge node mechanism	Availability	It accurately detects jamming attacks	The method was not tested in a real-world environment
[196]	Relies on channel and routing assignment and does not require additional hardware	Availability	It improves the packet ratio in IoT environments compared to existing methods	The method was not tested in a real-world environment
[197]	Based on SVM	Availability	It achieves a high detection rate	The method was tested using a simulation tool

**Table 6:** Summary of works focused on enhancing the security of the IIoT application layer.

Ref.	Algorithm	Resolved Issue	Provided Security Requirement	Dataset	Performance Metrics
[198]	Fuzzy pattern tree	malware	Integrity	Kaggle ${}^{a}$ and Vx-Heaven ${}^{b}$	97.0427% and 88.76% accuracies
[199]	LSTM	malware	Integrity	UNSW-NB15	70% accuracy
[200]	Fuzzy set theory and a new loss function	malware	Integrity	Drebin [201] and AndroZoo [202]	9% F1 score improvement
[203]	Fuzzy clustering	malware	Integrity	created from VirusShare ${}^{c}$, Kaggle, and Ransomware Tracker ${}^{d}$	VirusShare: 94.66%, Kaggle: 97.56%, Ransomware Tracker: 94.26% accuracies
[204]	Theoretical analysis	malware	Integrity	NA	NA
[205]	J48	ransomware	Confidentiality, authentication	VirusTotal	97.1% detection rate
[206]	*k*NN with DTW capability	ransomware	Confidentiality, authentication	VirusTotal	Window size 15: 94.27% accuracy, 95.65% recall, 89.19% precision, 92.31% F1 score
[207]	Decision tree and naïve Bayes	ransomware	Confidentiality, authentication	Custom	Packet-based (decision tree): 97.92% accuracy, 97.90 precision, recall, F1 score; flow-based (naïve Bayes): 97.08% accuracy, 97.72% precision, 97.71% recall and F1 score
[208]	Random forest	ransomware	Confidentiality, authentication	ransomware and malware-trusted	97.817% average F1 score of five splits
[209]	Logistic regression	ransomware	Confidentiality, authentication	created from VirusShare website	96.3% detection rate and 99.5% ROC curve
[210]	DNN	ransomware	Confidentiality, authentication	VirusTotal	93% accuracy
[211]	Practical analysis	ransomware	Confidentiality, authentication	Synthetic data	NA
[212]	Systemic analysis	ransomware	Confidentiality, authentication	Custom	NA

${}^{a}$ found at https://www.kaggle.com/c/malware-classification (accessed on 8 March 2023); ${}^{b}$ found at https://archive.ics.uci.edu/ml/datasets/Malware+static+and+dynamic+features+VxHeaven+and+Virus+Total (accessed on 12 March 2023); ${}^{c}$ found at https://www.virusshare.com/ (accessed on 12 March 2023); ${}^{d}$ found at https://ransomwaretracker.abuse.ch/ (accessed on 14 March 2023).

## Data Availability

Not applicable.

## Abbreviations

The following abbreviations are used in this manuscript:

ABC	Artificial Bee Colony
ACL	Access Control List
AES	Advanced Encryption Standard
AI	Artificial Intelligence
AMI	Advanced Metering Infrastructure
ANN	Artificial Neural Network
AP	Access Point
API	Application Programming Interface
AT&T	American Telephone and Telegraph
AUC	Area Under the Curve
B-LSTM	Bidirectional Long Short-Term Memory
CC	Cloud Computing
CFBPNN	Cascade Forward Back-Propagation Neural Network
CFS	Correlation-based Feature Selection
CIA	Confidentiality, Integrity, Availability
CPS	Cyber-Physical System
CSP	Cloud Service Provider
CSRF	Cross-Site Request Forgery
DDoS	Distributed Denial of Service
DL	Deep Learning
DNN	Deep Neural Network
DNS	Domain Name System
DoS	Denial of Service
DSI	Device-Side Injection
DSSS	Direct Sequence Spread Spectrum
DTW	Dynamic Time Warping
FDL	Federated Deep Learning
FHSS	Frequency Hopping Spread Spectrum
GDE	Global Detection Enactor
GE	General Electric
GM	Geometric Mean
GnuPG	GNU Privacy Guard
GRU	Gated Recurrent Unit
HAN	Home Area Network
HTTP	HyperText Transfer Protocol
IBM	International Business Machines
ICS	Industrial Control System
ICV	Intelligent Connected Vehicles
IDS	Intrusion Detection System
IIC	Industrial Internet Consortium
IIoT	Industrial Internet of Things
IIRA	Industrial Internet Reference Architecture
I/O	Input/Output
IoT	Internet of Things
IoV	Internet of Vehicles
IP	Internet Protocol
IPFS	Interplanetary File System
IPS	Intrusion Prevention System
*k*NN	*k*-Nearest Neighbors
LAE	Long short-term memory Autoencoder
LR	Logistic Regression
LSTM	Long Short-Term Memory
MC	Markov Chain
MCC	Mobile Cloud Computing
ML	Machine Learning
MLP	Multi-Layer Perceptron
NARX	Nonlinear Autoregressive network with eXogenous input
NFC	Near-field Communication
OLE	Object Linking and Embedding
OTA	Over-The-Air
PEC	Pervasive Edge Computing
PKI	Public Key Infrastructure
PLC	Program Logic Controller
PoV	Proof of Vote
PSO	Particle Swarm Optimization
PUF	Physically Unclonable Functions
RCE	Remote Code Execution
RFID	Radio Frequency Identification
RNN	Recurrent Neural Network
RPL	Routing Protocol for Low-power and lossy networks
RPT	Requesting Party Token
RSA	Rivest, Shamir, and Adleman
RTT	Round Trip Time
SCADA	Supervisory Control And Data Acquisition
SDN	Software-Defined Networking
SQL	Structured Query Language
SQLI	Structured Query Language Injection
SPOF	Single Point Of Failure
SMB	Server Message Block
SSI	Server-Side Injection
SSRF	Server-Side Request Forgery
SVM	Support Vector Machine
TEE	Trusted Execution Environment
TPM	Trusted Platform Module
TTP	Trusted Third Party
UMA	User-Managed Access
VCC	Vehicular Cloud Computing
VNF	Virtual Network Function
WAF	Web Application Firewall
Wi-Fi	Wireless Fidelity
WSN	Wireless Sensor Network
XGBoost	eXtreme Gradient Boosting
XML	eXtensible Markup Language
XSS	Cross-Site Scripting
